# Different packing motifs mediated by weak inter­actions and polymorphism in the crystal structures of five 2-(benzyl­idene)benzosuberone derivatives

**DOI:** 10.1107/S2056989019014245

**Published:** 2019-10-29

**Authors:** Lewis S. Seaman, Cristiane F. da Costa, Marcus V. N. de Souza, Solange M. S. V. Wardell, James L. Wardell, William T. A. Harrison

**Affiliations:** a Department of Chemistry, University of Aberdeen, Meston Walk, Aberdeen AB24 3UE, Scotland; b Fundação Oswaldo Cruz, Instituto de Tecnologia em Fármacos Manguinhos, 21041-250 Rio de Janeiro, RJ, Brazil; c CHEMSOL, 1 Harcourt Road, Aberdeen AB15 5NY, Scotland

**Keywords:** crystal structure, suberone, polymorphism

## Abstract

The syntheses and crystal structures of five 2-benzyl­idene-1-benzosuberone [1-benzosuberone is 6,7,8,9-tetra­hydro-5*H*-benzo[7]annulen-5-one] derivatives, *viz.* 2-(4-meth­oxy­benzyl­idene)-1-benzosuberone, C_19_H_18_O_2_, (I), 2-(4-eth­oxy­benzyl­idene)-1-benzosuberone, C_20_H_20_O_2_, (II), 2-(4-benzyl­benzyl­idene)-1-benzosuberone, C_25_H_22_O_2_, (III), 2-(4-chloro­benzyl­idene)-1-benzosuberone, C_18_H_15_ClO, (IV) and 2-(4-cyano­benzyl­idene)-1-benzosuberone, C_19_H_15_NO, (V), are described. The conformations of the benzosuberone fused six- plus seven-membered ring fragments are very similar in each case, but the dihedral angles between the fused benzene ring and the pendant benzene ring differ somewhat, with values of 23.79 (3) for (I), 24.60 (4) for (II), 33.72 (4) for (III), 29.93 (8) for (IV) and 21.81 (7)° for (V). Key features of the packing include pairwise C—H⋯O hydrogen bonds for (II) and (IV), and pairwise C—H⋯N hydrogen bonds for (V), which generate inversion dimers in each case. The packing for (I) and (III) feature C—H⋯O hydrogen bonds, which lead to [010] and [100] chains, respectively. Weak C—H⋯π inter­actions consolidate the structures and weak aromatic π–π stacking is seen in (II) [centroid–centroid separation = 3.8414 (7) Å] and (III) [3.9475 (7) Å]. A polymorph of (I) crystallized from a different solvent has been reported previously [Dimmock *et al.* (1999 ▸) *J. Med. Chem.* **42**, 1358–1366] in the same space group but with a packing motif based on inversion dimers resembling that seen in (IV) in the present study. The Hirshfeld surfaces and fingerprint plots for (I) and its polymorph are com­pared and structural features of the 2-benzyl­idene-1-benzosuberone family of phases are surveyed.

## Abstract 2

The title com­pounds, which differ in the substituent at the 4-position of the pendant benzene ring, show different packing motifs mediated by weak C—H⋯*X* (*X* = O or N) inter­actions. One of them is a polymorph of a known structure.

## Chemical context   

The structurally related 2-benzyl­idenebenzo­cycloalkanone com­pounds, *viz.* (*E*)-2-benzyl­idene-2,3-di­hydro-1*H*-inden-1-one (*n* = 1), (*E*)-2-benzyl­idene-1-tetra­lone (*n* = 2) and (*E*)-2-benzyl­idene-1-benzosuberone (*n* = 3), which differ with respect to the number of methyl­ene groups, *n*, in the alkanone ring fused to the benzene ring (see Scheme 1) have attracted attention in a number of areas. Their biological activities include anti­tumour (*e.g.* Gautam *et al.*, 2016 ▸: Dimmock *et al.*, 1999 ▸, 2002 ▸), anti­mycotic (Al-Nakib *et al.*, 1997 ▸) and anti­fungal (Gupta & Jain, 2015 ▸) properties. Their physical properties include nonlinear optical (Watson *et al.*, 1993 ▸) and UV hypsochromic shifts and fluorescence effects (Fodor *et al.*, 2011 ▸). It may be noted that these com­pounds can be considered as fused-ring analogues of chalcones (*i.e.* the ‘*n* = 0’ family), which might allow for ‘tuneable’ conformational control of the mol­ecule by changing the number of methyl­ene groups in the cyclo­alkanone ring (Dimmock *et al.*, 1999 ▸).

In continuation of our earlier reports of the crystal structures and Hirshfeld surface analyses of a number of (*E*)-2-benzyl­idene-2,3-di­hydro-1*H*-inden-1-one derivatives (Baddeley *et al.*, 2017*a* ▸) and (*E*)-2-benzyl­idene-1-tetra­lone (Baddeley *et al.*, 2017*b* ▸), we now describe the syntheses and crystal structures of 2-(4-meth­oxy­benzyl­idene)-1-benzosuberone, (I), 2-(4-eth­oxy­benzyl­idene)-1-benzosuberone, (II), 2-(4-benzyl­benzyl­idene)-1-benzosuberone, (III), 2-(4-chloro­benzyl­idene)-1-benzo­suberone, (IV), and 2-(4-cyano­benzyl­idene)-1-benzosuberone, (V) (see Scheme 2).
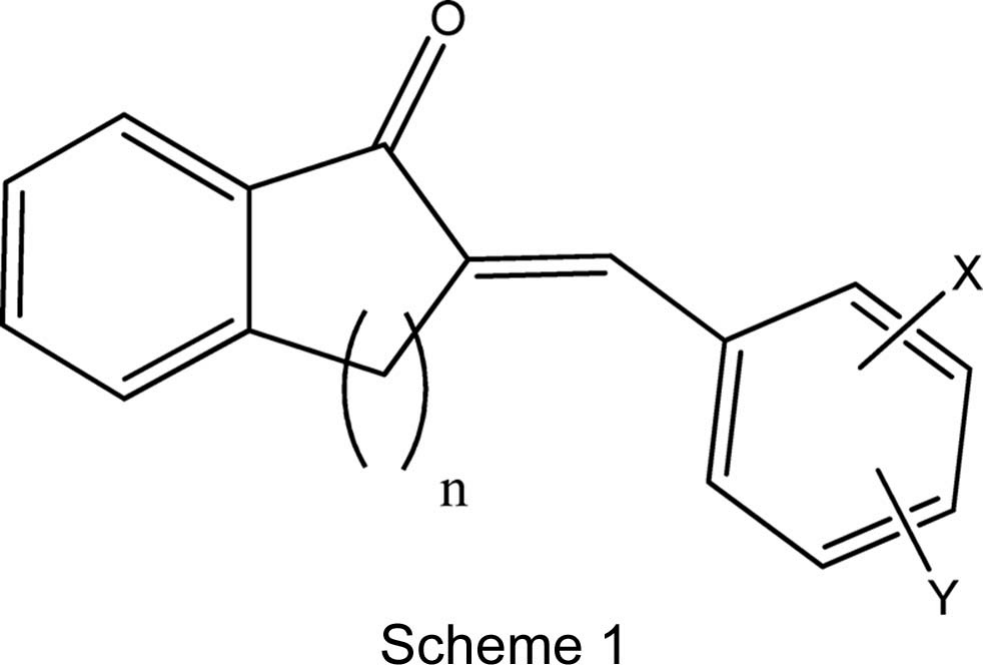
 
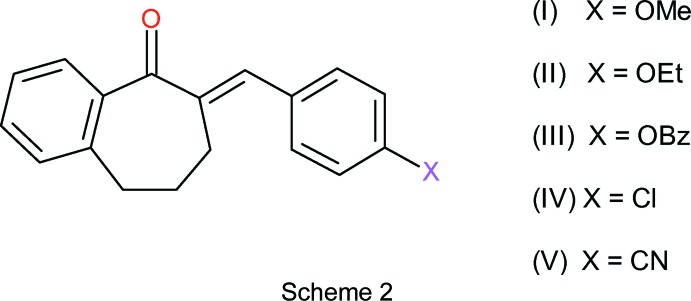


## Structural commentary   

**Figure 1:**
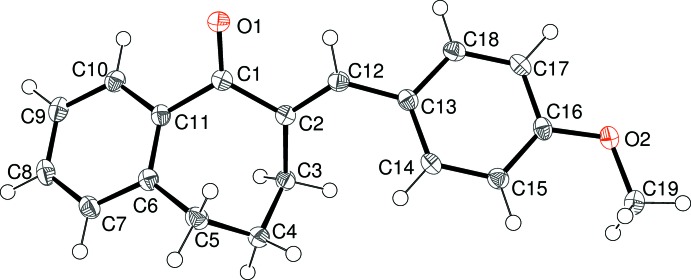
The mol­ecular structure of (I), showing 50% probability displacement ellipsoids.

**Figure 2:**
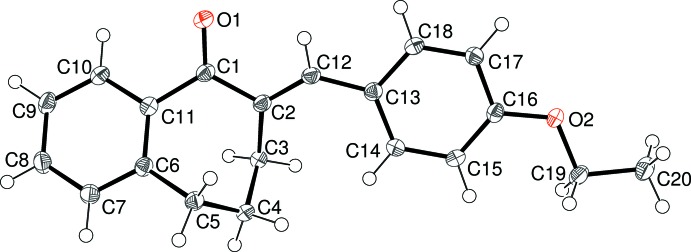
The mol­ecular structure of (II), showing 50% probability displacement ellipsoids.

**Figure 3:**
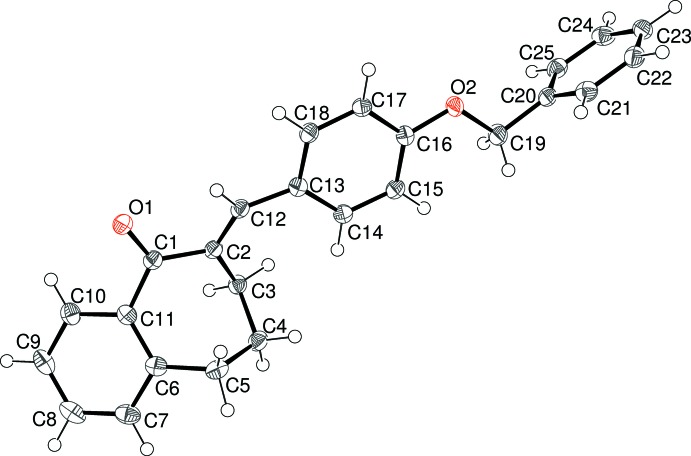
The mol­ecular structure of (III), showing 50% probability displacement ellipsoids.

**Figure 4:**
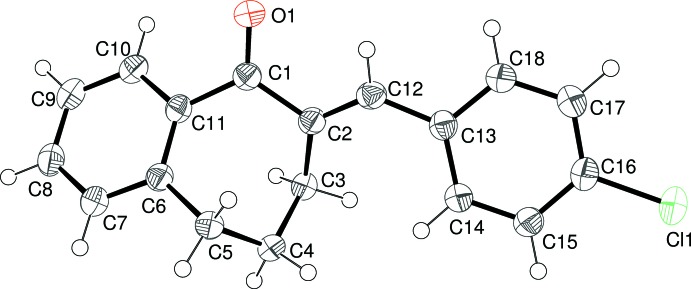
The mol­ecular structure of (IV), showing 50% probability displacement ellipsoids.

**Figure 5:**
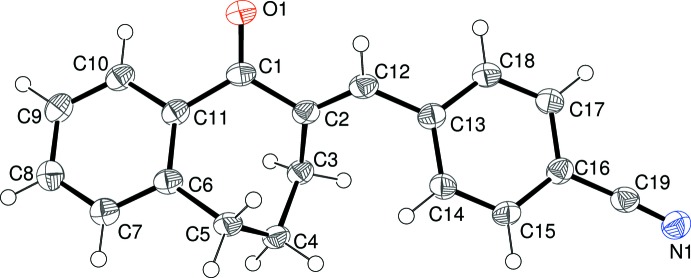
The mol­ecular structure of (V), showing 50% probability displacement ellipsoids.

**Figure 6:**
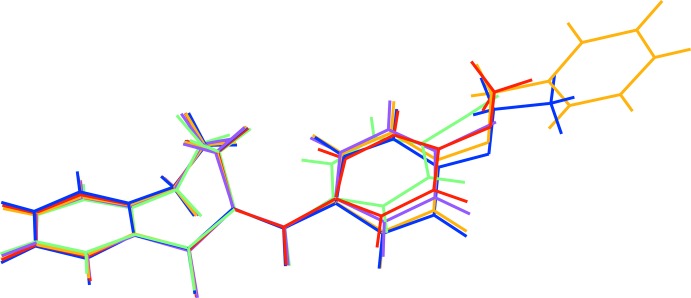
Overlay plot for (I)–(V), with (I) red, (II) blue, (III) orange, (IV) purple and (V) green.

The mol­ecular structures of (I)–(V) are shown in Figs. 1–5 ▸ ▸ ▸ ▸ ▸, respectively. Each mol­ecule is the expected product arising from the base-catalysed condensation reaction between 1-benzosuberone and the appropriate 4-substituted benzaldehyde derivative (see *Experimental* section). The conformations of the benzosuberone fragments in (I)–(V) are almost identical, as shown by the overlay plot generated with *QMOL* (Gans & Shalloway, 2001 ▸) shown in Fig. 6 ▸. The seven-membered ring, which is conformationally constrained by being fused to the C6–C11 benzene ring and by the presence of the *sp* ^2^-hybridized atoms C1 and C2, at least approximates to a boat conformation; in the case of (I), atoms C3/C4/C6/C11 are roughly coplanar (r.m.s. deviation = 0.095 Å), with C5 as the prow [deviation = 0.6139 (15) Å] and C1 and C2 as the stern [deviations = 1.0114 (16) and 1.0154 (16) Å, respectively]. This conformation results in a substantial degree of twist about the C11—C1 bond [C10—C11—C1=O1 = 36.06 (14)°] and O1 deviates from the C6–C11 benzene-ring plane by 0.7212 (17) Å. The corresponding data for the seven-membered rings in (II)–(V) are very similar to those for (I) and are not stated here.

The dihedral angles between the C6–C11 fused benzene ring and the C13–C18 pendant benzene ring are clustered in a ∼12° range, with values of 23.79 (3) for (I), 24.60 (4) for (II), 33.72 (4) for (III), 29.93 (8) for (IV) and 21.81 (7)° for (V). A com­parison of the C1—C2—C12—C13 and C2—C12—C13—C14 torsion angles for (I) [−179.67 (10) and −33.81 (17)°, respectively] indicates that the twisting largely occurs about the C12—C13 bond, and the same conclusion can be drawn for (II)–(V).

For (I), the C19 atom of the meth­oxy group is close to coplanar with its attached benzene ring [deviation = 0.1079 (19) Å] and for (II) the eth­oxy group has an extended conformation [C16—O2—C19—C20 = 178.58 (10)°]. For (III), an additional dihedral angle between the C13–C18 benzene ring and the terminal C20–C25 benzene ring of 78.78 (3)° is observed. Otherwise, the geometrical data for (I)–(V) are unexceptional and similar to those for related com­pounds (Dimmock *et al.*, 1999 ▸, 2002 ▸).

It may be noted that a polymorph of (I) [Cambridge Structural Database (CSD; Groom *et al.*, 2016 ▸) refcode VENQUA; Dimmock *et al.*, 1999 ▸] has been reported in the same space group, *i.e.* *P*2_1_/*c*; VENQUA was recrystallized from methanol solution rather than ethanol for (I). The bond lengths and angles in (I) and VENQUA are very similar, although there is a ∼10° difference in the dihedral angle between the benzene rings [value for VENQUA = 35.88 (11)°, calculated with *PLATON* (Spek, 2009 ▸)]; for an overlay plot of (I) and VENQUA, see the supporting information.

## Supra­molecular features   

**Figure 7:**
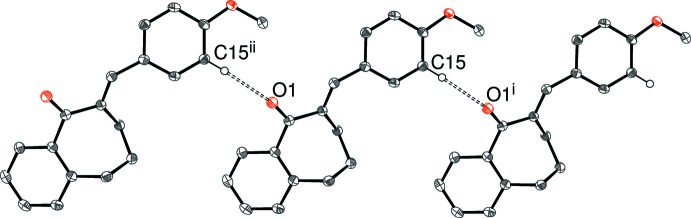
Fragment of the crystal structure of (I), showing part of a [010] chain linked by C15—H15⋯O1 hydrogen bonds. [Symmetry codes: (i) *x*, *y* − 1, *z*; (ii) *x*, *y* + 1, *z*.]

There are obviously no classical hydrogen bonds in these structures and, in each case, just one C—H group can be identified as the donor for a weak hydrogen bond with atom O1 as the acceptor in (I)–(IV) and atom N1 in (V); geometrical data for these inter­actions are listed in Tables 1 ▸–5 ▸ ▸ ▸ ▸ and illustrated in Figs. 7 ▸–11 ▸ ▸ ▸ ▸. All the structures also feature weak C—H⋯π inter­actions with either the fused or pendant benzene rings as acceptors, but (II) and (III) are the only structures to display weak aromatic π–π stacking, in both cases between inversion-related C13–C18 rings. For (II), the centroid–centroid separation is 3.8414 (7) Å and the slippage is 1.72 Å; equivalent data for (III) are 3.9475 (7) and 1.89 Å, respectively.

The packing motifs for the extended structures of (I) and (III) are infinite C—H⋯O hydrogen-bonded chains, which propagate in the [010] and [100] directions, respectively. In each case, adjacent mol­ecules are related only by unit-cell translational symmetry and a *C*(8) graph-set motif results for both structures with the methyne group (C15—H15, *ortho* to the 4-substituent) involved as the donor.

**Figure 8:**
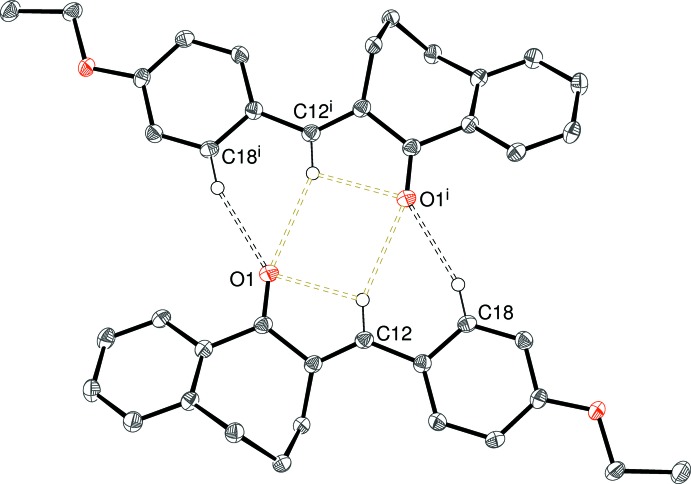
Fragment of the crystal structure of (II), showing inversion dimers linked by pairs of C18—H18⋯O1 hydrogen bonds. [Symmetry code: (i) −*x* + 1, −*y* + 1, −*z* + 1.]

The packing motifs for (II) and (IV) feature inversion dimers. In (II), C18—H18 (*meta* to the 4-substituent) is the donor group and 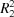
(14) loops arise. In this motif, C12—H12 is ‘sandwiched’ between the donor and acceptor and the H12⋯O1 separation of 2.60 Å (see Fig. 8 ▸) is borderline to be regarded as a directional bond. The donor group in (IV) is C10—H10 in the fused benzene ring, which generates an 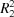
(10) loop. The only possible inter­action involving the Cl atom is a long contact from C8—H8, with H⋯Cl = 2.93 Å. The presence of the cyano group in (V) allows for the formation of pairwise C—H⋯N hydrogen bonds and an 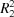
(10) graph-set motif arises; the shortest H⋯O contact in (V) is 2.72 Å.

Rather than the *C*(8) chains arising from C15—H15⋯O1 hydrogen bonds seen in (I), the packing for VENQUA (see above) features inversion dimers built from pairwise C10—H10⋯O1 inter­actions, which are very similar to those seen in 4-chloro derivative (IV) in the present study. It may be noted that the density of VENQUA (ρ = 1.368 Mg m^−3^) is significantly greater than that of (I) (ρ = 1.284 Mg m^−3^), suggesting that the former might be the more stable polymorph if the ‘rational packing rule’ (Kitaigorodskii, 1961 ▸) is applicable in this case.

In order to gain further insight into these different packing motifs, the Hirshfeld surfaces and fingerprint plots for (I) and VENQUA were calculated using *CrystalExplorer* (Turner *et al.*, 2017 ▸), following the approach recently described by Tan *et al.* (2019 ▸). The Hirshfeld surface for (I) (see supporting information) shows the expected large red spots (close contacts) in the vicinity of H15 and O1 corresponding to the C15—H15⋯O1 inter­action noted above, but there is little if any evidence of close contacts in the vicinity of H19*A* and H19*C* corresponding to the C—H⋯π contacts listed in Table 1 ▸. The surface for VENQUA (see supporting information) shows red spots in the vicinity of H10 and O1 corresponding to the C10—H10⋯O1 hydrogen bond and H2*A* (our numbering scheme) corresponding to a C3—H2*A*⋯π inter­action (H⋯π = 2.69 Å) to the centroid of the C6–C11 benzene ring, but there are also probably spurious features close to H8 and H17 corresponding to a short H⋯H contact of 2.07 Å between these atoms, which possibly arose because the H atoms of the C19 methyl group in VENQUA were geometrically placed and not treated using a rotating-group model. Notwithstanding this, the fingerprint plots for (I) and VENQUA (see supporting information) decom­posed into the different percentage contact types (Table 6 ▸) are almost identical; H⋯H (van der Waals) contacts dominate both structures, followed by C⋯H/H⋯C and then O⋯H/H⋯O. The percentage contributions of the other contact types are negligible.

## Database survey   

A survey of the Cambridge Structural Database (CSD; Groom *et al.*, 2016 ▸) revealed 167 structures incorporating a 1-benzosuberone fragment but only 20 hits when an exocyclic C=C double bond at the 2-position was added to the search structure. The key papers reporting the structures of closely related, differently substituted, 2-benzyl­idene-1-benzosuberones are Dimmock *et al.* (1999 ▸, 2002 ▸). The hydrogen-bond data for (I)–(V) and the 12 structures reported in the two papers by Dimmock *et al.* are summarized in Table 7 ▸. The most frequently observed motif is the centrosymmetric 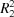
(10) loop involving C10—H10 as the donor group, but there are many others involving different C—H groups as donor and we see no obvious connection to the nature and position of the substituent(s) on the remote benzene ring. There are no structures in which the fused and pendant benzene rings tend towards being perpendicular (dihedral angle > 60°).

The fact that (I) and VENQUA have similar conformations but distinct packing motifs mediated by different C—H⋯O inter­actions to the same acceptor O atom may be com­pared with the fascinating recent survey of weak-inter­action polymorphs by Lo Presti (2018 ▸). He concluded that weak hydrogen bonds and solvent effects may play an important kinetic role in promoting polymorph formation (after all, something has to favour a situation where the lowest-energy packing motif is not adopted) but they do not play a dominant energetic role in polymorph formation and that the overall energy balance between dispersive (attractive) and repulsive inter­actions is the most important consideration.

## Synthesis and crystallization   

Compounds (I)–(V) were obtained from the reaction of 1-benzosuberone (1 mmol) with the appropriate 4-substituted benzaldehyde (1 mmol) in ethanol (5 ml) treated with an ethano­lic solution of sodium hydroxide (30 mg in 5 ml ethanol). After stirring for 3–4 h at room temperature, each reaction mixture was cooled to 0 °C and the precipitated solid was recovered by filtration and rinsing with ice-cold ethanol. Recrystallization from ethanol solution at room temperature yielded colourless blocks [(I), (III) and (V)] and plates [(II) and (IV)]. Spectroscopic data for (I)–(V) are available as supporting information.

## Refinement   

Crystal data, data collection and structure refinement details are summarized in Table 8 ▸. All H atoms were located geometrically (C—H = 0.95–0.99 Å) and refined as riding atoms, with *U*_iso_(H) = 1.2*U*_eq_(C) or 1.5*U*_eq_(methyl C). The methyl groups in (I) and (II) were allowed to rotate, but not to tip, to best fit the electron density.

## Supplementary Material

Crystal structure: contains datablock(s) I, II, III, IV, V, global. DOI: 10.1107/S2056989019014245/eb2024sup1.cif

Structure factors: contains datablock(s) I. DOI: 10.1107/S2056989019014245/eb2024Isup2.hkl

Structure factors: contains datablock(s) II. DOI: 10.1107/S2056989019014245/eb2024IIsup3.hkl

Structure factors: contains datablock(s) III. DOI: 10.1107/S2056989019014245/eb2024IIIsup4.hkl

Structure factors: contains datablock(s) IV. DOI: 10.1107/S2056989019014245/eb2024IVsup5.hkl

Structure factors: contains datablock(s) V. DOI: 10.1107/S2056989019014245/eb2024Vsup6.hkl

CCDC references: 1960122, 1960121, 1960120, 1960119, 1960118, 1960118, 1960119, 1960120, 1960121, 1960122

Additional supporting information:  crystallographic information; 3D view; checkCIF report

## Figures and Tables

**Figure 9:**
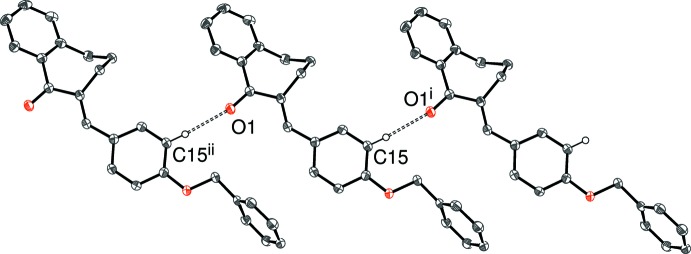
Fragment of the crystal structure of (III), showing part of a [100] chain linked by C15—H15⋯O1 hydrogen bonds. [Symmetry codes: (i) *x* + 1, *y*, *z*; (ii) *x* − 1, *y*, *z*.]

**Figure 10:**
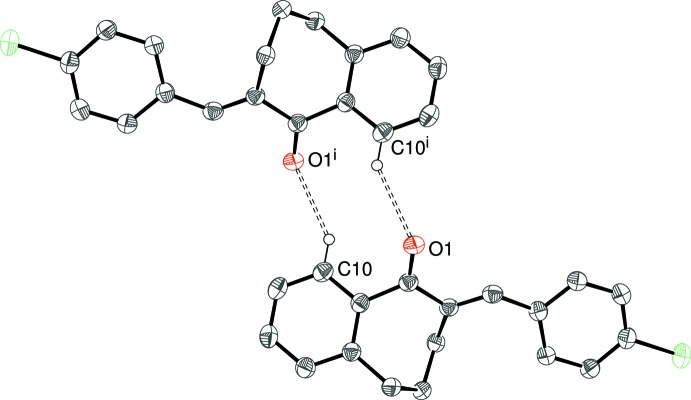
Fragment of the crystal structure of (IV), showing inversion dimers linked by pairs of C10—H10⋯O1 hydrogen bonds. [Symmetry code: (i) −*x*, −*y* + 1, −*z* + 1.]

**Figure 11:**
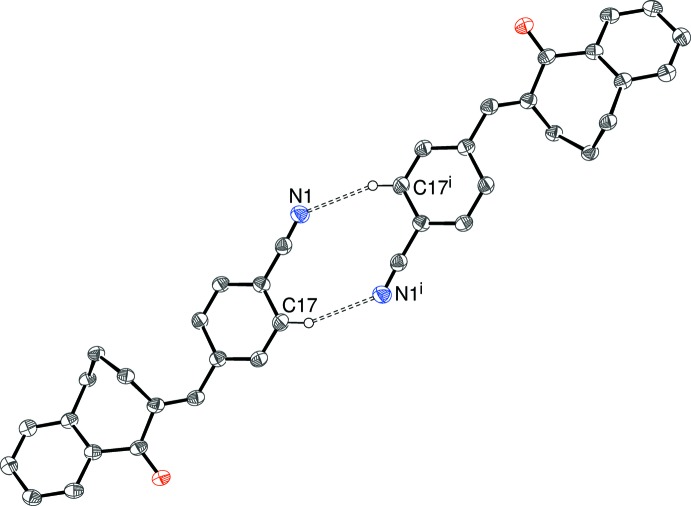
Fragment of the crystal structure of (V), showing inversion dimers linked by pairs of C17—H17⋯N1 hydrogen bonds. [Symmetry code: −*x*, −*y* + 1, −*z* + 1.]

**Table 1:** Hydrogen-bond geometry (Å, °) for (I) *Cg*1 and *Cg*2 are the centroids of the C6–C11 and C13–C18 rings, respectively.

*D*—H⋯*A*	*D*—H	H⋯*A*	*D*⋯*A*	*D*—H⋯*A*
C15—H15⋯O1^i^	0.95	2.35	3.2971 (14)	176
C19—H19*A*⋯*Cg*1^ii^	0.98	2.76	3.6165 (13)	146
C19—H19*C*⋯*Cg*2^iii^	0.98	2.74	3.6029 (13)	147

Symmetry codes: (i) 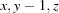; (ii) 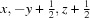; (iii) 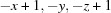.

**Table 2:** Hydrogen-bond geometry (Å, °) for (II) *Cg*1 and *Cg*2 are the centroids of the C6–C11 and C13–C18 rings, respectively.

*D*—H⋯*A*	*D*—H	H⋯*A*	*D*⋯*A*	*D*—H⋯*A*
C18—H18⋯O1^i^	0.95	2.36	3.2653 (14)	159
C4—H4*B*⋯*Cg*1^ii^	0.98	2.72	3.6429 (13)	155
C19—H19*A*⋯*Cg*2^ii^	0.98	2.71	3.5969 (13)	149

Symmetry codes: (i) 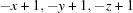; (ii) 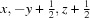.

**Table 3:** Hydrogen-bond geometry (Å, °) for (III) *Cg*3 is the centroid of the C20–C25 ring.

*D*—H⋯*A*	*D*—H	H⋯*A*	*D*⋯*A*	*D*—H⋯*A*
C15—H15⋯O1^i^	0.95	2.40	3.3477 (13)	176
C18—H18⋯*Cg*3^ii^	0.95	2.64	3.5147 (13)	153

Symmetry codes: (i) 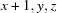; (ii) 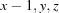.

**Table 4:** Hydrogen-bond geometry (Å, °) for (IV) *Cg*1 is the centroid of the C6–C11 ring.

*D*—H⋯*A*	*D*—H	H⋯*A*	*D*⋯*A*	*D*—H⋯*A*
C10—H10⋯O1^i^	0.95	2.50	3.319 (2)	145
C3—H3*A*⋯*Cg*1^ii^	0.99	2.83	3.572 (2)	132

Symmetry codes: (i) 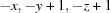; (ii) 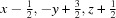.

**Table 5:** Hydrogen-bond geometry (Å, °) for (V) *Cg*1 is the centroid of the C6–C11 ring.

*D*—H⋯*A*	*D*—H	H⋯*A*	*D*⋯*A*	*D*—H⋯*A*
C17—H17⋯N1^i^	0.95	2.54	3.438 (2)	157
C3—H3*A*⋯*Cg*1^ii^	0.99	2.84	3.6730 (16)	142
C8—H8⋯*Cg*1^iii^	0.95	2.88	3.7868 (17)	161

Symmetry codes: (i) 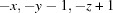; (ii) 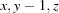; (iii) 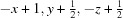.

**Table 6:** Fingerprint contact percentages for (I) and VENQUA

Contact type	(I)	VENQUA
H⋯H	54.8	55.3
C⋯H/H⋯C	28.1	29.2
O—H/H⋯O	15.3	14.5
C⋯C	1.1	0.0
C⋯O/O⋯C	0.8	0.8
O⋯O	0.0	0.2

**Table 7:** Summary of the C—H⋯O and C—H⋯N hydrogen bonds and packing motifs for 2-(benzyl­idene)benzosuberone derivatives

Code/refcode	Substituent(*s*)	Space group	φ	Donor atom(*s*)	Packing motif
(I)	4-OMe	*P*2_1_/*c*	23.79 (3)	C15	*C*(8) chain
(II)	4-OEt	*P*2_1_/*c*	24.60 (4)	C18	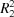 (14) loop
(III)	4-OBz	*P* 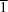	33.72 (4)	C15	*C*(8) chain
(IV)	4-Cl	*P*2_1_/*n*	29.93 (8)	C10	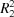 (10) loop
(V)	4-CN	*P*2_1_/*c*	21.81 (7)	C17	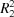 (10) loop
VENQOU	4-Me	*P*2_1_/*n*	29.72 (11)	C10	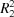 (10) loop
VENQUA	4-OMe	*P*2_1_/*n*	35.88 (11)	C10	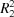 (10) loop
VENSIQ	4-NMe_2_	*P*2_1_/*n*	29.43 (11)	C10	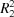 (10) loop
XUGXOM	2-NO_2_	*P*2_1_/*a*	27.56 (6)	C17	*C*(5) chain
VENREL	3-NO_2_	*P* 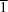	18.54 (9)	C7,C14,C16	double chain
VENRIP	4-NO_2_	*P* 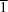	45.32 (9)	C9,C15	sheet
XUGYED	2-Cl	*P*2_1_/*c*	28.40 (19)	C14	*C*(7) chain
XUGXUS	3,4-Cl	*P*2_1_/*c*	39.01 (16)	C15	*C*(8) chain
XUGYAZ	2,4-Cl	*P*2_1_/*c*	30.54 (12)	C14	*C*(7) chain
XUGYUT	2-OMe	*P*2_1_	25.82 (17)	None	–
XUGYON	3,4-OMe	*P* 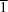	23.48 (9)	C8,C15	sheet
XUGYIH	3,4,5-OMe	*P*2_1_/*n*	35.08 (10)	C7	*C*(6) chain

Notes: packing analyses were carried out using *PLATON* (Spek, 2009 ▸); φ is the dihedral angle between the C6–C11 and C13–C18 benzene rings; for the ‘VEN’ refcode family, see Dimmock *et al.* (1999 ▸); for the ‘XUG’ family, see Dimmock *et al.* (2002 ▸); the donor atom labels correspond to our atom numbering scheme.

**Table:** 

	(I)	(II)	(III)
Crystal data			
Chemical formula	C_19_H_18_O_2_	C_20_H_20_O_2_	C_25_H_22_O_2_
*M*_r_	278.33	292.36	354.42
Crystal system, space group	Monoclinic, *P*2_1_/*c*	Monoclinic, *P*2_1_/*c*	Triclinic, *P* 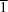
Temperature (K)	100	100	100
*a*, *b*, *c* (Å)	10.9171 (3), 9.1262 (2), 15.2539 (3)	12.6208 (2), 14.99690 (17), 8.39151 (12)	9.2870 (2), 9.8727 (2), 12.2944 (3)
α, β, γ (°)	90, 108.618 (3), 90	90, 108.6814 (17), 90	67.098 (3), 81.472 (2), 61.989 (3)
*V* (Å^3^)	1440.24 (6)	1504.60 (4)	915.92 (5)
*Z*	4	4	2
Radiation type	Mo *K*α	Cu *K*α	Cu *K*α
μ (mm^−1^)	0.08	0.64	0.63
Crystal size (mm)	0.20 × 0.15 × 0.05	0.20 × 0.11 × 0.03	0.17 × 0.11 × 0.04

Data collection			
Diffractometer	XtaLAB AFC12 (RCD3): Kappa single CCD	XtaLAB AFC11 (RCD3): quarter-chi single CCD	XtaLAB AFC11 (RCD3): quarter-chi single CCD
Absorption correction	Multi-scan (*CrysAlis PRO*; Rigaku, 2017 ▸)	Gaussian (*CrysAlis PRO*; Rigaku, 2017 ▸)	Gaussian (*CrysAlis PRO*; Rigaku, 2017 ▸)
*T*_min_, *T*_max_	0.877, 1.000	0.772, 1.000	0.781, 1.000
No. of measured, independent and observed [*I* > 2σ(*I*)] reflections	16988, 3296, 2843	9197, 2704, 2486	29818, 3336, 3073
*R*_int_	0.033	0.024	0.036
(sin θ/λ)_max_ (Å^−1^)	0.649	0.602	0.602

Refinement			
*R*[*F* ^2^ > 2σ(*F* ^2^)], *wR*(*F* ^2^), *S*	0.037, 0.093, 1.03	0.034, 0.092, 1.04	0.032, 0.080, 1.07
No. of reflections	3296	2704	3336
No. of parameters	191	201	245
H-atom treatment	H-atom parameters constrained	H-atom parameters constrained	H-atom parameters constrained
Δρ_max_, Δρ_min_ (e Å^−3^)	0.25, −0.18	0.25, −0.20	0.19, −0.16

**Table:** 

	(IV)	(V)
Crystal data		
Chemical formula	C_18_H_15_ClO	C_19_H_15_NO
*M*_r_	282.75	273.32
Crystal system, space group	Monoclinic, *P*2_1_/*n*	Monoclinic, *P*2_1_/*c*
Temperature (K)	100	100
*a*, *b*, *c* (Å)	10.6273 (5), 11.6191 (4), 12.1114 (5)	12.4725 (4), 7.1718 (2), 15.9983 (5)
α, β, γ (°)	90, 108.777 (4), 90	90, 106.120 (3), 90
*V* (Å^3^)	1415.92 (11)	1374.79 (8)
*Z*	4	4
Radiation type	Cu *K*α	Cu *K*α
μ (mm^−1^)	2.31	0.64
Crystal size (mm)	0.28 × 0.20 × 0.03	0.17 × 0.10 × 0.03

Data collection		
Diffractometer	XtaLAB AFC11 (RCD3): quarter-chi single CCD	XtaLAB AFC11 (RCD3): quarter-chi single CCD
Absorption correction	Multi-scan (*CrysAlis PRO*; Rigaku, 2017 ▸)	Gaussian (*CrysAlis PRO*; Rigaku, 2017 ▸)
*T*_min_, *T*_max_	0.722, 1.000	0.895, 1.000
No. of measured, independent and observed [*I* > 2σ(*I*)] reflections	11747, 2568, 2203	9732, 2511, 2302
*R*_int_	0.073	0.059
(sin θ/λ)_max_ (Å^−1^)	0.602	0.602

Refinement		
*R*[*F* ^2^ > 2σ(*F* ^2^)], *wR*(*F* ^2^), *S*	0.055, 0.165, 1.11	0.068, 0.181, 1.06
No. of reflections	2568	2511
No. of parameters	181	190
H-atom treatment	H-atom parameters constrained	H-atom parameters constrained
Δρ_max_, Δρ_min_ (e Å^−3^)	0.32, −0.41	0.49, −0.32

Computer programs: *CrysAlis PRO* (Rigaku, 2017 ▸), *SHELXS97* (Sheldrick, 2008 ▸), *SHELXL2014* (Sheldrick, 2015 ▸), *ORTEP-3* (Farrugia, 2012 ▸) and *publCIF* (Westrip, 2010 ▸).

## supplementary crystallographic information

### 6-(4-Methoxybenzylidene)-6,7,8,9-tetrahydro-5H-benzo[7]annulen-5-one (I) Crystal data

**Table:**

C_19_H_18_O_2_	*F*(000) = 592
*M**_r_* = 278.33	*D*_x_ = 1.284 Mg m^−^^3^
Monoclinic, *P*2_1_/*c*	Mo *K*α radiation, λ = 0.71073 Å
*a* = 10.9171 (3) Å	Cell parameters from 6994 reflections
*b* = 9.1262 (2) Å	θ = 3.6–30.6°
*c* = 15.2539 (3) Å	µ = 0.08 mm^−^^1^
β = 108.618 (3)°	*T* = 100 K
*V* = 1440.24 (6) Å^3^	Block, colourless
*Z* = 4	0.20 × 0.15 × 0.05 mm

### 6-(4-Methoxybenzylidene)-6,7,8,9-tetrahydro-5H-benzo[7]annulen-5-one (I) Data collection

**Table:**

XtaLAB AFC12 (RCD3): Kappa single CCD diffractometer	3296 independent reflections
Radiation source: Rotating-anode X-ray tube	2843 reflections with *I* > 2σ(*I*)
Mirror monochromator	*R*_int_ = 0.033
ω scans	θ_max_ = 27.5°, θ_min_ = 2.6°
Absorption correction: multi-scan (CrysAlis PRO; Rigaku, 2017)	*h* = −13→14
*T*_min_ = 0.877, *T*_max_ = 1.000	*k* = −11→11
16988 measured reflections	*l* = −19→19

### 6-(4-Methoxybenzylidene)-6,7,8,9-tetrahydro-5H-benzo[7]annulen-5-one (I) Refinement

**Table:**

Refinement on *F*^2^	Primary atom site location: structure-invariant direct methods
Least-squares matrix: full	Hydrogen site location: inferred from neighbouring sites
*R*[*F*^2^ > 2σ(*F*^2^)] = 0.037	H-atom parameters constrained
*wR*(*F*^2^) = 0.093	*w* = 1/[σ^2^(*F*_o_^2^) + (0.0435*P*)^2^ + 0.435*P*] where *P* = (*F*_o_^2^ + 2*F*_c_^2^)/3
*S* = 1.03	(Δ/σ)_max_ < 0.001
3296 reflections	Δρ_max_ = 0.25 e Å^−^^3^
191 parameters	Δρ_min_ = −0.18 e Å^−^^3^
0 restraints	

### 6-(4-Methoxybenzylidene)-6,7,8,9-tetrahydro-5H-benzo[7]annulen-5-one (I) Special details

**Table:**

Geometry. All esds (except the esd in the dihedral angle between two l.s. planes) are estimated using the full covariance matrix. The cell esds are taken into account individually in the estimation of esds in distances, angles and torsion angles; correlations between esds in cell parameters are only used when they are defined by crystal symmetry. An approximate (isotropic) treatment of cell esds is used for estimating esds involving l.s. planes.

### 6-(4-Methoxybenzylidene)-6,7,8,9-tetrahydro-5H-benzo[7]annulen-5-one (I) Fractional atomic coordinates and isotropic or equivalent isotropic displacement parameters (Å^2^)

**Table:**

	*x*	*y*	*z*	*U*_iso_*/*U*_eq_	
C1	0.21223 (10)	0.67551 (12)	0.33073 (7)	0.0185 (2)	
C2	0.25289 (10)	0.51984 (12)	0.35285 (7)	0.0176 (2)	
C3	0.26700 (10)	0.42493 (12)	0.27568 (7)	0.0192 (2)	
H3A	0.3201	0.3384	0.3032	0.023*	
H3B	0.3146	0.4809	0.2413	0.023*	
C4	0.13905 (11)	0.37140 (12)	0.20672 (7)	0.0235 (2)	
H4A	0.1543	0.3392	0.1491	0.028*	
H4B	0.1077	0.2858	0.2333	0.028*	
C5	0.03505 (11)	0.49088 (12)	0.18348 (8)	0.0228 (2)	
H5A	−0.0381	0.4586	0.1296	0.027*	
H5B	0.0022	0.5024	0.2365	0.027*	
C6	0.08193 (10)	0.63762 (12)	0.16175 (7)	0.0187 (2)	
C7	0.03877 (11)	0.69424 (13)	0.07205 (7)	0.0220 (2)	
H7	−0.0170	0.6369	0.0237	0.026*	
C8	0.07549 (11)	0.83208 (13)	0.05212 (8)	0.0237 (2)	
H8	0.0461	0.8677	−0.0096	0.028*	
C9	0.15527 (11)	0.91857 (13)	0.12212 (8)	0.0227 (2)	
H9	0.1795	1.0139	0.1087	0.027*	
C10	0.19928 (10)	0.86472 (12)	0.21178 (7)	0.0199 (2)	
H10	0.2534	0.9237	0.2600	0.024*	
C11	0.16467 (10)	0.72442 (12)	0.23167 (7)	0.0178 (2)	
C12	0.28541 (10)	0.48266 (12)	0.44276 (7)	0.0184 (2)	
H12	0.2785	0.5598	0.4828	0.022*	
C13	0.32958 (10)	0.34296 (12)	0.48884 (7)	0.0179 (2)	
C14	0.28868 (10)	0.20616 (12)	0.44938 (7)	0.0186 (2)	
H14	0.2300	0.2016	0.3881	0.022*	
C15	0.33124 (10)	0.07653 (12)	0.49704 (7)	0.0192 (2)	
H15	0.3014	−0.0152	0.4689	0.023*	
C16	0.41828 (10)	0.08253 (12)	0.58673 (7)	0.0182 (2)	
C17	0.45955 (10)	0.21776 (12)	0.62786 (7)	0.0205 (2)	
H17	0.5189	0.2220	0.6889	0.025*	
C18	0.41448 (11)	0.34552 (12)	0.58016 (7)	0.0204 (2)	
H18	0.4414	0.4371	0.6096	0.024*	
C19	0.41639 (11)	−0.17646 (12)	0.60092 (8)	0.0220 (2)	
H19A	0.3224	−0.1777	0.5874	0.033*	
H19B	0.4555	−0.2544	0.6452	0.033*	
H19C	0.4370	−0.1924	0.5436	0.033*	
O1	0.21902 (8)	0.76586 (9)	0.39153 (5)	0.0253 (2)	
O2	0.46646 (7)	−0.03768 (8)	0.63967 (5)	0.02142 (18)	

### 6-(4-Methoxybenzylidene)-6,7,8,9-tetrahydro-5H-benzo[7]annulen-5-one (I) Atomic displacement parameters (Å^2^)

**Table:**

	*U*^11^	*U*^22^	*U*^33^	*U*^12^	*U*^13^	*U*^23^
C1	0.0175 (5)	0.0198 (5)	0.0189 (5)	−0.0002 (4)	0.0067 (4)	−0.0003 (4)
C2	0.0171 (5)	0.0181 (5)	0.0178 (5)	−0.0003 (4)	0.0058 (4)	−0.0001 (4)
C3	0.0236 (6)	0.0186 (5)	0.0166 (5)	0.0020 (4)	0.0081 (4)	0.0007 (4)
C4	0.0305 (6)	0.0199 (6)	0.0183 (5)	−0.0024 (5)	0.0054 (5)	−0.0002 (4)
C5	0.0215 (6)	0.0247 (6)	0.0204 (5)	−0.0040 (4)	0.0041 (4)	0.0002 (4)
C6	0.0165 (5)	0.0211 (5)	0.0192 (5)	0.0020 (4)	0.0065 (4)	0.0011 (4)
C7	0.0195 (5)	0.0265 (6)	0.0186 (5)	0.0036 (4)	0.0041 (4)	0.0004 (4)
C8	0.0246 (6)	0.0283 (6)	0.0192 (5)	0.0083 (5)	0.0083 (4)	0.0070 (4)
C9	0.0250 (6)	0.0203 (6)	0.0260 (6)	0.0048 (4)	0.0127 (5)	0.0057 (4)
C10	0.0196 (5)	0.0194 (5)	0.0220 (5)	0.0027 (4)	0.0086 (4)	−0.0001 (4)
C11	0.0174 (5)	0.0192 (5)	0.0182 (5)	0.0032 (4)	0.0076 (4)	0.0015 (4)
C12	0.0187 (5)	0.0193 (5)	0.0181 (5)	0.0003 (4)	0.0070 (4)	−0.0014 (4)
C13	0.0177 (5)	0.0213 (5)	0.0159 (5)	0.0009 (4)	0.0071 (4)	0.0008 (4)
C14	0.0178 (5)	0.0233 (6)	0.0142 (5)	−0.0003 (4)	0.0042 (4)	0.0008 (4)
C15	0.0200 (5)	0.0204 (5)	0.0175 (5)	−0.0015 (4)	0.0065 (4)	−0.0002 (4)
C16	0.0179 (5)	0.0218 (5)	0.0163 (5)	0.0027 (4)	0.0076 (4)	0.0037 (4)
C17	0.0193 (5)	0.0271 (6)	0.0140 (5)	0.0004 (4)	0.0037 (4)	0.0003 (4)
C18	0.0222 (5)	0.0218 (5)	0.0176 (5)	−0.0011 (4)	0.0071 (4)	−0.0024 (4)
C19	0.0241 (6)	0.0199 (5)	0.0226 (5)	0.0015 (4)	0.0081 (4)	0.0041 (4)
O1	0.0349 (5)	0.0208 (4)	0.0187 (4)	0.0036 (3)	0.0066 (3)	−0.0019 (3)
O2	0.0239 (4)	0.0209 (4)	0.0177 (4)	0.0025 (3)	0.0043 (3)	0.0037 (3)

### 6-(4-Methoxybenzylidene)-6,7,8,9-tetrahydro-5H-benzo[7]annulen-5-one (I) Geometric parameters (Å, º)

**Table:**

C1—O1	1.2254 (13)	C9—H9	0.9500
C1—C2	1.4945 (15)	C10—C11	1.3955 (15)
C1—C11	1.5003 (14)	C10—H10	0.9500
C2—C12	1.3458 (15)	C12—C13	1.4614 (15)
C2—C3	1.5085 (14)	C12—H12	0.9500
C3—C4	1.5356 (15)	C13—C14	1.3959 (15)
C3—H3A	0.9900	C13—C18	1.4056 (15)
C3—H3B	0.9900	C14—C15	1.3887 (15)
C4—C5	1.5319 (16)	C14—H14	0.9500
C4—H4A	0.9900	C15—C16	1.3956 (15)
C4—H4B	0.9900	C15—H15	0.9500
C5—C6	1.5078 (15)	C16—O2	1.3642 (12)
C5—H5A	0.9900	C16—C17	1.3926 (15)
C5—H5B	0.9900	C17—C18	1.3793 (15)
C6—C7	1.3962 (15)	C17—H17	0.9500
C6—C11	1.4021 (15)	C18—H18	0.9500
C7—C8	1.3832 (16)	C19—O2	1.4304 (13)
C7—H7	0.9500	C19—H19A	0.9800
C8—C9	1.3885 (17)	C19—H19B	0.9800
C8—H8	0.9500	C19—H19C	0.9800
C9—C10	1.3869 (15)		
			
O1—C1—C2	121.79 (10)	C8—C9—H9	120.3
O1—C1—C11	118.68 (10)	C9—C10—C11	120.47 (11)
C2—C1—C11	119.51 (9)	C9—C10—H10	119.8
C12—C2—C1	115.63 (9)	C11—C10—H10	119.8
C12—C2—C3	126.23 (10)	C10—C11—C6	120.42 (10)
C1—C2—C3	117.81 (9)	C10—C11—C1	117.47 (10)
C2—C3—C4	114.83 (9)	C6—C11—C1	121.99 (9)
C2—C3—H3A	108.6	C2—C12—C13	130.49 (10)
C4—C3—H3A	108.6	C2—C12—H12	114.8
C2—C3—H3B	108.6	C13—C12—H12	114.8
C4—C3—H3B	108.6	C14—C13—C18	117.46 (10)
H3A—C3—H3B	107.5	C14—C13—C12	124.18 (9)
C5—C4—C3	112.15 (9)	C18—C13—C12	118.30 (10)
C5—C4—H4A	109.2	C15—C14—C13	121.92 (10)
C3—C4—H4A	109.2	C15—C14—H14	119.0
C5—C4—H4B	109.2	C13—C14—H14	119.0
C3—C4—H4B	109.2	C14—C15—C16	119.28 (10)
H4A—C4—H4B	107.9	C14—C15—H15	120.4
C6—C5—C4	113.86 (9)	C16—C15—H15	120.4
C6—C5—H5A	108.8	O2—C16—C17	115.97 (9)
C4—C5—H5A	108.8	O2—C16—C15	124.19 (10)
C6—C5—H5B	108.8	C17—C16—C15	119.84 (10)
C4—C5—H5B	108.8	C18—C17—C16	120.12 (10)
H5A—C5—H5B	107.7	C18—C17—H17	119.9
C7—C6—C11	118.05 (10)	C16—C17—H17	119.9
C7—C6—C5	120.80 (10)	C17—C18—C13	121.34 (10)
C11—C6—C5	121.07 (9)	C17—C18—H18	119.3
C8—C7—C6	121.41 (11)	C13—C18—H18	119.3
C8—C7—H7	119.3	O2—C19—H19A	109.5
C6—C7—H7	119.3	O2—C19—H19B	109.5
C7—C8—C9	120.18 (10)	H19A—C19—H19B	109.5
C7—C8—H8	119.9	O2—C19—H19C	109.5
C9—C8—H8	119.9	H19A—C19—H19C	109.5
C10—C9—C8	119.45 (11)	H19B—C19—H19C	109.5
C10—C9—H9	120.3	C16—O2—C19	116.31 (8)
			
O1—C1—C2—C12	6.07 (15)	O1—C1—C11—C10	36.06 (14)
C11—C1—C2—C12	−175.42 (9)	C2—C1—C11—C10	−142.50 (10)
O1—C1—C2—C3	−167.73 (10)	O1—C1—C11—C6	−140.03 (11)
C11—C1—C2—C3	10.78 (14)	C2—C1—C11—C6	41.42 (14)
C12—C2—C3—C4	110.47 (12)	C1—C2—C12—C13	−179.67 (10)
C1—C2—C3—C4	−76.47 (12)	C3—C2—C12—C13	−6.47 (19)
C2—C3—C4—C5	40.48 (13)	C2—C12—C13—C14	−33.81 (17)
C3—C4—C5—C6	46.53 (12)	C2—C12—C13—C18	148.98 (11)
C4—C5—C6—C7	110.60 (11)	C18—C13—C14—C15	−1.16 (15)
C4—C5—C6—C11	−72.74 (13)	C12—C13—C14—C15	−178.38 (10)
C11—C6—C7—C8	−0.30 (16)	C13—C14—C15—C16	−0.55 (15)
C5—C6—C7—C8	176.46 (10)	C14—C15—C16—O2	−179.87 (9)
C6—C7—C8—C9	−1.05 (17)	C14—C15—C16—C17	1.11 (15)
C7—C8—C9—C10	0.97 (16)	O2—C16—C17—C18	−179.03 (9)
C8—C9—C10—C11	0.46 (16)	C15—C16—C17—C18	0.07 (15)
C9—C10—C11—C6	−1.83 (15)	C16—C17—C18—C13	−1.85 (16)
C9—C10—C11—C1	−177.98 (9)	C14—C13—C18—C17	2.36 (15)
C7—C6—C11—C10	1.73 (15)	C12—C13—C18—C17	179.75 (9)
C5—C6—C11—C10	−175.02 (10)	C17—C16—O2—C19	174.98 (9)
C7—C6—C11—C1	177.70 (9)	C15—C16—O2—C19	−4.07 (14)
C5—C6—C11—C1	0.95 (15)		

### 6-(4-Methoxybenzylidene)-6,7,8,9-tetrahydro-5H-benzo[7]annulen-5-one (I) Hydrogen-bond geometry (Å, º)

**Table:**

*D*—H···*A*	*D*—H	H···*A*	*D*···*A*	*D*—H···*A*
C15—H15···O1^i^	0.95	2.35	3.2971 (14)	176
C19—H19*A*···*Cg*1^ii^	0.98	2.76	3.6165 (13)	146
C19—H19*C*···*Cg*2^iii^	0.98	2.74	3.6029 (13)	147

Symmetry codes: (i) *x*, *y*−1, *z*; (ii) *x*, −*y*+1/2, *z*+1/2; (iii) −*x*+1, −*y*, −*z*+1.

### 6-(4-Ethoxybenzylidene)-6,7,8,9-tetrahydro-5H-benzo[7]annulen-5-one (II) Crystal data

**Table:**

C_20_H_20_O_2_	*F*(000) = 624
*M**_r_* = 292.36	*D*_x_ = 1.291 Mg m^−^^3^
Monoclinic, *P*2_1_/*c*	Cu *K*α radiation, λ = 1.54184 Å
*a* = 12.6208 (2) Å	Cell parameters from 4578 reflections
*b* = 14.99690 (17) Å	θ = 3.7–69.7°
*c* = 8.39151 (12) Å	µ = 0.64 mm^−^^1^
β = 108.6814 (17)°	*T* = 100 K
*V* = 1504.60 (4) Å^3^	Plate, colourless
*Z* = 4	0.20 × 0.11 × 0.03 mm

### 6-(4-Ethoxybenzylidene)-6,7,8,9-tetrahydro-5H-benzo[7]annulen-5-one (II) Data collection

**Table:**

XtaLAB AFC11 (RCD3): quarter-chi single CCD diffractometer	2704 independent reflections
Radiation source: Rotating-anode X-ray tube	2486 reflections with *I* > 2σ(*I*)
Mirror monochromator	*R*_int_ = 0.024
ω scans	θ_max_ = 68.3°, θ_min_ = 3.7°
Absorption correction: gaussian (CrysAlis PRO; Rigaku, 2017)	*h* = −14→15
*T*_min_ = 0.772, *T*_max_ = 1.000	*k* = −18→12
9197 measured reflections	*l* = −9→9

### 6-(4-Ethoxybenzylidene)-6,7,8,9-tetrahydro-5H-benzo[7]annulen-5-one (II) Refinement

**Table:**

Refinement on *F*^2^	Hydrogen site location: inferred from neighbouring sites
Least-squares matrix: full	H-atom parameters constrained
*R*[*F*^2^ > 2σ(*F*^2^)] = 0.034	*w* = 1/[σ^2^(*F*_o_^2^) + (0.0491*P*)^2^ + 0.4554*P*] where *P* = (*F*_o_^2^ + 2*F*_c_^2^)/3
*wR*(*F*^2^) = 0.092	(Δ/σ)_max_ < 0.001
*S* = 1.04	Δρ_max_ = 0.25 e Å^−^^3^
2704 reflections	Δρ_min_ = −0.20 e Å^−^^3^
201 parameters	Extinction correction: SHELXL2014 (Sheldrick, 2015), Fc^*^=kFc[1+0.001xFc^2^λ^3^/sin(2θ)]^-1/4^
0 restraints	Extinction coefficient: 0.0019 (3)
Primary atom site location: structure-invariant direct methods	

### 6-(4-Ethoxybenzylidene)-6,7,8,9-tetrahydro-5H-benzo[7]annulen-5-one (II) Special details

**Table:**

Geometry. All esds (except the esd in the dihedral angle between two l.s. planes) are estimated using the full covariance matrix. The cell esds are taken into account individually in the estimation of esds in distances, angles and torsion angles; correlations between esds in cell parameters are only used when they are defined by crystal symmetry. An approximate (isotropic) treatment of cell esds is used for estimating esds involving l.s. planes.

### 6-(4-Ethoxybenzylidene)-6,7,8,9-tetrahydro-5H-benzo[7]annulen-5-one (II) Fractional atomic coordinates and isotropic or equivalent isotropic displacement parameters (Å^2^)

**Table:**

	*x*	*y*	*z*	*U*_iso_*/*U*_eq_	
C1	0.29882 (10)	0.41642 (7)	0.43555 (14)	0.0187 (3)	
C2	0.34088 (9)	0.37739 (8)	0.60870 (14)	0.0181 (3)	
C3	0.27266 (9)	0.30342 (8)	0.64912 (14)	0.0183 (3)	
H3A	0.3219	0.2668	0.7414	0.022*	
H3B	0.2438	0.2646	0.5492	0.022*	
C4	0.17406 (9)	0.33720 (8)	0.70118 (14)	0.0196 (3)	
H4A	0.2021	0.3603	0.8178	0.024*	
H4B	0.1226	0.2870	0.6990	0.024*	
C5	0.10987 (10)	0.41143 (8)	0.58327 (14)	0.0203 (3)	
H5A	0.0363	0.4193	0.6001	0.024*	
H5B	0.1517	0.4680	0.6155	0.024*	
C6	0.09109 (10)	0.39437 (7)	0.39855 (14)	0.0187 (3)	
C7	−0.01602 (10)	0.37634 (8)	0.28964 (15)	0.0218 (3)	
H7	−0.0757	0.3683	0.3342	0.026*	
C8	−0.03708 (10)	0.36985 (8)	0.11691 (15)	0.0239 (3)	
H8	−0.1104	0.3564	0.0451	0.029*	
C9	0.04853 (10)	0.38295 (8)	0.04913 (15)	0.0233 (3)	
H9	0.0337	0.3808	−0.0692	0.028*	
C10	0.15569 (10)	0.39914 (8)	0.15573 (14)	0.0201 (3)	
H10	0.2145	0.4080	0.1097	0.024*	
C11	0.17889 (10)	0.40265 (7)	0.33013 (14)	0.0183 (3)	
C12	0.43722 (9)	0.41219 (8)	0.71077 (14)	0.0182 (3)	
H12	0.4688	0.4565	0.6587	0.022*	
C13	0.50252 (10)	0.39471 (7)	0.88640 (14)	0.0183 (3)	
C14	0.46487 (10)	0.35195 (8)	1.00672 (15)	0.0207 (3)	
H14	0.3911	0.3284	0.9737	0.025*	
C15	0.53262 (10)	0.34296 (8)	1.17341 (15)	0.0209 (3)	
H15	0.5052	0.3134	1.2523	0.025*	
C16	0.64063 (10)	0.37742 (7)	1.22393 (14)	0.0186 (3)	
C17	0.67988 (9)	0.42064 (8)	1.10640 (14)	0.0193 (3)	
H17	0.7537	0.4441	1.1399	0.023*	
C18	0.61171 (10)	0.42937 (8)	0.94187 (14)	0.0186 (3)	
H18	0.6393	0.4597	0.8639	0.022*	
C19	0.67659 (10)	0.33225 (8)	1.51117 (15)	0.0229 (3)	
H19A	0.6531	0.2700	1.4798	0.028*	
H19B	0.6122	0.3655	1.5247	0.028*	
C20	0.77366 (11)	0.33441 (9)	1.67216 (15)	0.0271 (3)	
H20A	0.7515	0.3069	1.7626	0.041*	
H20B	0.7960	0.3964	1.7018	0.041*	
H20C	0.8367	0.3014	1.6569	0.041*	
O1	0.36138 (7)	0.45674 (6)	0.37458 (10)	0.0264 (2)	
O2	0.71428 (7)	0.37337 (6)	1.38349 (10)	0.0217 (2)	

### 6-(4-Ethoxybenzylidene)-6,7,8,9-tetrahydro-5H-benzo[7]annulen-5-one (II) Atomic displacement parameters (Å^2^)

**Table:**

	*U*^11^	*U*^22^	*U*^33^	*U*^12^	*U*^13^	*U*^23^
C1	0.0219 (6)	0.0195 (6)	0.0159 (6)	−0.0012 (4)	0.0076 (5)	−0.0007 (4)
C2	0.0192 (6)	0.0212 (6)	0.0155 (6)	0.0017 (4)	0.0077 (5)	0.0005 (4)
C3	0.0182 (6)	0.0208 (6)	0.0155 (5)	−0.0006 (4)	0.0049 (4)	0.0011 (4)
C4	0.0195 (6)	0.0250 (6)	0.0154 (5)	−0.0014 (5)	0.0071 (5)	0.0005 (4)
C5	0.0196 (6)	0.0251 (6)	0.0171 (6)	0.0020 (5)	0.0072 (5)	−0.0011 (4)
C6	0.0204 (6)	0.0187 (5)	0.0171 (6)	0.0027 (4)	0.0059 (5)	0.0012 (4)
C7	0.0203 (6)	0.0255 (6)	0.0203 (6)	0.0027 (5)	0.0073 (5)	−0.0004 (5)
C8	0.0197 (6)	0.0297 (6)	0.0195 (6)	0.0019 (5)	0.0023 (5)	−0.0015 (5)
C9	0.0260 (6)	0.0279 (6)	0.0144 (6)	0.0027 (5)	0.0045 (5)	−0.0007 (5)
C10	0.0233 (6)	0.0213 (6)	0.0169 (6)	0.0008 (5)	0.0081 (5)	0.0007 (4)
C11	0.0205 (6)	0.0173 (5)	0.0167 (6)	0.0004 (4)	0.0056 (5)	0.0002 (4)
C12	0.0184 (6)	0.0207 (6)	0.0176 (6)	0.0010 (4)	0.0087 (5)	0.0007 (4)
C13	0.0188 (6)	0.0198 (5)	0.0170 (6)	0.0010 (4)	0.0067 (5)	−0.0008 (4)
C14	0.0169 (6)	0.0258 (6)	0.0195 (6)	−0.0022 (5)	0.0063 (5)	0.0001 (5)
C15	0.0216 (6)	0.0254 (6)	0.0175 (6)	−0.0014 (5)	0.0090 (5)	0.0022 (4)
C16	0.0202 (6)	0.0200 (6)	0.0149 (6)	0.0026 (4)	0.0049 (5)	−0.0009 (4)
C17	0.0174 (6)	0.0220 (6)	0.0187 (6)	−0.0008 (4)	0.0060 (5)	−0.0012 (4)
C18	0.0205 (6)	0.0199 (6)	0.0173 (6)	0.0003 (4)	0.0089 (5)	0.0002 (4)
C19	0.0277 (6)	0.0260 (6)	0.0160 (6)	−0.0015 (5)	0.0084 (5)	0.0017 (5)
C20	0.0329 (7)	0.0287 (7)	0.0174 (6)	−0.0010 (5)	0.0049 (5)	0.0016 (5)
O1	0.0244 (5)	0.0366 (5)	0.0183 (4)	−0.0081 (4)	0.0071 (3)	0.0043 (4)
O2	0.0207 (4)	0.0296 (5)	0.0137 (4)	−0.0018 (3)	0.0041 (3)	0.0025 (3)

### 6-(4-Ethoxybenzylidene)-6,7,8,9-tetrahydro-5H-benzo[7]annulen-5-one (II) Geometric parameters (Å, º)

**Table:**

C1—O1	1.2286 (14)	C10—C11	1.3985 (16)
C1—C2	1.4971 (16)	C10—H10	0.9500
C1—C11	1.5027 (16)	C12—C13	1.4635 (16)
C2—C12	1.3481 (17)	C12—H12	0.9500
C2—C3	1.5079 (16)	C13—C14	1.4014 (16)
C3—C4	1.5311 (15)	C13—C18	1.4053 (17)
C3—H3A	0.9900	C14—C15	1.3927 (16)
C3—H3B	0.9900	C14—H14	0.9500
C4—C5	1.5365 (16)	C15—C16	1.3909 (17)
C4—H4A	0.9900	C15—H15	0.9500
C4—H4B	0.9900	C16—O2	1.3650 (14)
C5—C6	1.5130 (16)	C16—C17	1.3968 (16)
C5—H5A	0.9900	C17—C18	1.3794 (16)
C5—H5B	0.9900	C17—H17	0.9500
C6—C7	1.3944 (17)	C18—H18	0.9500
C6—C11	1.4074 (16)	C19—O2	1.4426 (14)
C7—C8	1.3908 (17)	C19—C20	1.5056 (17)
C7—H7	0.9500	C19—H19A	0.9900
C8—C9	1.3868 (18)	C19—H19B	0.9900
C8—H8	0.9500	C20—H20A	0.9800
C9—C10	1.3832 (18)	C20—H20B	0.9800
C9—H9	0.9500	C20—H20C	0.9800
			
O1—C1—C2	121.41 (10)	C11—C10—H10	119.4
O1—C1—C11	118.88 (10)	C10—C11—C6	119.69 (11)
C2—C1—C11	119.65 (10)	C10—C11—C1	117.07 (10)
C12—C2—C1	115.63 (10)	C6—C11—C1	123.24 (10)
C12—C2—C3	127.28 (10)	C2—C12—C13	132.29 (11)
C1—C2—C3	117.09 (10)	C2—C12—H12	113.9
C2—C3—C4	113.30 (9)	C13—C12—H12	113.9
C2—C3—H3A	108.9	C14—C13—C18	116.96 (11)
C4—C3—H3A	108.9	C14—C13—C12	126.59 (11)
C2—C3—H3B	108.9	C18—C13—C12	116.32 (10)
C4—C3—H3B	108.9	C15—C14—C13	121.81 (11)
H3A—C3—H3B	107.7	C15—C14—H14	119.1
C3—C4—C5	111.41 (9)	C13—C14—H14	119.1
C3—C4—H4A	109.3	C16—C15—C14	119.77 (11)
C5—C4—H4A	109.3	C16—C15—H15	120.1
C3—C4—H4B	109.3	C14—C15—H15	120.1
C5—C4—H4B	109.3	O2—C16—C15	125.13 (10)
H4A—C4—H4B	108.0	O2—C16—C17	115.41 (10)
C6—C5—C4	114.49 (10)	C15—C16—C17	119.46 (11)
C6—C5—H5A	108.6	C18—C17—C16	120.15 (11)
C4—C5—H5A	108.6	C18—C17—H17	119.9
C6—C5—H5B	108.6	C16—C17—H17	119.9
C4—C5—H5B	108.6	C17—C18—C13	121.83 (10)
H5A—C5—H5B	107.6	C17—C18—H18	119.1
C7—C6—C11	118.32 (11)	C13—C18—H18	119.1
C7—C6—C5	120.34 (11)	O2—C19—C20	106.86 (10)
C11—C6—C5	121.17 (10)	O2—C19—H19A	110.3
C8—C7—C6	121.21 (11)	C20—C19—H19A	110.3
C8—C7—H7	119.4	O2—C19—H19B	110.3
C6—C7—H7	119.4	C20—C19—H19B	110.3
C9—C8—C7	120.24 (11)	H19A—C19—H19B	108.6
C9—C8—H8	119.9	C19—C20—H20A	109.5
C7—C8—H8	119.9	C19—C20—H20B	109.5
C10—C9—C8	119.24 (11)	H20A—C20—H20B	109.5
C10—C9—H9	120.4	C19—C20—H20C	109.5
C8—C9—H9	120.4	H20A—C20—H20C	109.5
C9—C10—C11	121.14 (11)	H20B—C20—H20C	109.5
C9—C10—H10	119.4	C16—O2—C19	117.69 (9)
			
O1—C1—C2—C12	20.55 (16)	O1—C1—C11—C10	27.78 (16)
C11—C1—C2—C12	−162.35 (10)	C2—C1—C11—C10	−149.40 (11)
O1—C1—C2—C3	−158.66 (11)	O1—C1—C11—C6	−151.50 (11)
C11—C1—C2—C3	18.45 (15)	C2—C1—C11—C6	31.33 (16)
C12—C2—C3—C4	97.96 (14)	C1—C2—C12—C13	177.92 (11)
C1—C2—C3—C4	−82.94 (12)	C3—C2—C12—C13	−3.0 (2)
C2—C3—C4—C5	45.35 (13)	C2—C12—C13—C14	−17.9 (2)
C3—C4—C5—C6	42.50 (13)	C2—C12—C13—C18	166.41 (12)
C4—C5—C6—C7	110.32 (12)	C18—C13—C14—C15	−0.80 (17)
C4—C5—C6—C11	−74.39 (14)	C12—C13—C14—C15	−176.51 (11)
C11—C6—C7—C8	−2.33 (17)	C13—C14—C15—C16	0.32 (18)
C5—C6—C7—C8	173.09 (11)	C14—C15—C16—O2	179.45 (11)
C6—C7—C8—C9	−1.20 (18)	C14—C15—C16—C17	−0.06 (17)
C7—C8—C9—C10	2.45 (18)	O2—C16—C17—C18	−179.24 (10)
C8—C9—C10—C11	−0.12 (18)	C15—C16—C17—C18	0.32 (17)
C9—C10—C11—C6	−3.44 (17)	C16—C17—C18—C13	−0.85 (17)
C9—C10—C11—C1	177.26 (11)	C14—C13—C18—C17	1.07 (17)
C7—C6—C11—C10	4.59 (16)	C12—C13—C18—C17	177.23 (10)
C5—C6—C11—C10	−170.79 (10)	C15—C16—O2—C19	−1.70 (16)
C7—C6—C11—C1	−176.16 (11)	C17—C16—O2—C19	177.83 (10)
C5—C6—C11—C1	8.46 (17)	C20—C19—O2—C16	178.58 (10)

### 6-(4-Ethoxybenzylidene)-6,7,8,9-tetrahydro-5H-benzo[7]annulen-5-one (II) Hydrogen-bond geometry (Å, º)

**Table:**

*D*—H···*A*	*D*—H	H···*A*	*D*···*A*	*D*—H···*A*
C18—H18···O1^i^	0.95	2.36	3.2653 (14)	159
C4—H4*B*···*Cg*1^ii^	0.98	2.72	3.6429 (13)	155
C19—H19*A*···*Cg*2^ii^	0.98	2.71	3.5969 (13)	149

Symmetry codes: (i) −*x*+1, −*y*+1, −*z*+1; (ii) *x*, −*y*+1/2, *z*+1/2.

### 2-(4-Benzylbenzylidene)-6,7,8,9-tetrahydro-5H-benzo[7]annulen-5-one (III) Crystal data

**Table:**

C_25_H_22_O_2_	*Z* = 2
*M**_r_* = 354.42	*F*(000) = 376
Triclinic, *P*1	*D*_x_ = 1.285 Mg m^−^^3^
*a* = 9.2870 (2) Å	Cu *K*α radiation, λ = 1.54184 Å
*b* = 9.8727 (2) Å	Cell parameters from 19041 reflections
*c* = 12.2944 (3) Å	θ = 3.9–70.3°
α = 67.098 (3)°	µ = 0.63 mm^−^^1^
β = 81.472 (2)°	*T* = 100 K
γ = 61.989 (3)°	Block, colourless
*V* = 915.92 (5) Å^3^	0.17 × 0.11 × 0.04 mm

### 2-(4-Benzylbenzylidene)-6,7,8,9-tetrahydro-5H-benzo[7]annulen-5-one (III) Data collection

**Table:**

XtaLAB AFC11 (RCD3): quarter-chi single CCD diffractometer	3336 independent reflections
Radiation source: Rotating-anode X-ray tube	3073 reflections with *I* > 2σ(*I*)
Mirror monochromator	*R*_int_ = 0.036
ω scans	θ_max_ = 68.2°, θ_min_ = 3.9°
Absorption correction: gaussian (CrysAlis PRO; Rigaku, 2017)	*h* = −11→11
*T*_min_ = 0.781, *T*_max_ = 1.000	*k* = −11→11
29818 measured reflections	*l* = −14→14

### 2-(4-Benzylbenzylidene)-6,7,8,9-tetrahydro-5H-benzo[7]annulen-5-one (III) Refinement

**Table:**

Refinement on *F*^2^	Hydrogen site location: inferred from neighbouring sites
Least-squares matrix: full	H-atom parameters constrained
*R*[*F*^2^ > 2σ(*F*^2^)] = 0.032	*w* = 1/[σ^2^(*F*_o_^2^) + (0.0343*P*)^2^ + 0.2601*P*] where *P* = (*F*_o_^2^ + 2*F*_c_^2^)/3
*wR*(*F*^2^) = 0.080	(Δ/σ)_max_ < 0.001
*S* = 1.07	Δρ_max_ = 0.19 e Å^−^^3^
3336 reflections	Δρ_min_ = −0.16 e Å^−^^3^
245 parameters	Extinction correction: SHELXL2014 (Sheldrick, 2015), Fc^*^=kFc[1+0.001xFc^2^λ^3^/sin(2θ)]^-1/4^
0 restraints	Extinction coefficient: 0.0027 (4)
Primary atom site location: structure-invariant direct methods	

### 2-(4-Benzylbenzylidene)-6,7,8,9-tetrahydro-5H-benzo[7]annulen-5-one (III) Special details

**Table:**

Geometry. All esds (except the esd in the dihedral angle between two l.s. planes) are estimated using the full covariance matrix. The cell esds are taken into account individually in the estimation of esds in distances, angles and torsion angles; correlations between esds in cell parameters are only used when they are defined by crystal symmetry. An approximate (isotropic) treatment of cell esds is used for estimating esds involving l.s. planes.

### 2-(4-Benzylbenzylidene)-6,7,8,9-tetrahydro-5H-benzo[7]annulen-5-one (III) Fractional atomic coordinates and isotropic or equivalent isotropic displacement parameters (Å^2^)

**Table:**

	*x*	*y*	*z*	*U*_iso_*/*U*_eq_	
C1	−0.05094 (13)	0.41786 (13)	0.37210 (9)	0.0206 (2)	
C2	0.11044 (12)	0.39278 (12)	0.40498 (9)	0.0201 (2)	
C3	0.15112 (13)	0.53459 (13)	0.34098 (9)	0.0218 (2)	
H3A	0.2299	0.5293	0.3905	0.026*	
H3B	0.0507	0.6397	0.3286	0.026*	
C4	0.22435 (13)	0.53175 (14)	0.22121 (9)	0.0253 (2)	
H4A	0.3404	0.4482	0.2340	0.030*	
H4B	0.2204	0.6406	0.1732	0.030*	
C5	0.13332 (14)	0.49174 (14)	0.15296 (9)	0.0261 (2)	
H5A	0.1675	0.5174	0.0702	0.031*	
H5B	0.1661	0.3720	0.1880	0.031*	
C6	−0.05030 (13)	0.58461 (13)	0.15292 (9)	0.0232 (2)	
C7	−0.14024 (15)	0.70291 (13)	0.04850 (10)	0.0286 (3)	
H7	−0.0846	0.7283	−0.0221	0.034*	
C8	−0.30954 (15)	0.78425 (14)	0.04574 (10)	0.0311 (3)	
H8	−0.3685	0.8652	−0.0262	0.037*	
C9	−0.39279 (14)	0.74770 (14)	0.14755 (11)	0.0295 (3)	
H9	−0.5087	0.8033	0.1457	0.035*	
C10	−0.30610 (13)	0.62955 (13)	0.25218 (10)	0.0254 (2)	
H10	−0.3632	0.6033	0.3218	0.030*	
C11	−0.13575 (13)	0.54864 (12)	0.25643 (9)	0.0214 (2)	
C12	0.20322 (12)	0.24621 (13)	0.48767 (9)	0.0203 (2)	
H12	0.1549	0.1733	0.5162	0.024*	
C13	0.36457 (13)	0.17816 (13)	0.54185 (9)	0.0208 (2)	
C14	0.48036 (13)	0.23711 (13)	0.49726 (9)	0.0232 (2)	
H14	0.4568	0.3274	0.4241	0.028*	
C15	0.62886 (13)	0.16712 (13)	0.55709 (9)	0.0239 (2)	
H15	0.7051	0.2099	0.5253	0.029*	
C16	0.66527 (12)	0.03382 (13)	0.66400 (9)	0.0214 (2)	
C17	0.55458 (13)	−0.03095 (12)	0.70815 (9)	0.0213 (2)	
H17	0.5800	−0.1236	0.7799	0.026*	
C18	0.40818 (13)	0.03976 (13)	0.64743 (9)	0.0211 (2)	
H18	0.3344	−0.0065	0.6779	0.025*	
C19	0.92156 (13)	0.02302 (14)	0.68813 (10)	0.0291 (3)	
H19A	0.8709	0.1402	0.6798	0.035*	
H19B	0.9567	0.0162	0.6096	0.035*	
C20	1.06575 (13)	−0.07617 (13)	0.77387 (9)	0.0230 (2)	
C21	1.18414 (13)	−0.22910 (13)	0.77295 (10)	0.0251 (2)	
H21	1.1719	−0.2709	0.7190	0.030*	
C22	1.31964 (13)	−0.32064 (13)	0.85007 (10)	0.0266 (2)	
H22	1.3985	−0.4259	0.8499	0.032*	
C23	1.34081 (13)	−0.25957 (14)	0.92737 (10)	0.0273 (3)	
H23	1.4350	−0.3216	0.9791	0.033*	
C24	1.22388 (14)	−0.10747 (14)	0.92885 (10)	0.0283 (3)	
H24	1.2376	−0.0652	0.9819	0.034*	
C25	1.08673 (13)	−0.01680 (13)	0.85305 (10)	0.0257 (2)	
H25	1.0063	0.0868	0.8552	0.031*	
O1	−0.11776 (9)	0.33551 (9)	0.43769 (6)	0.02637 (19)	
O2	0.80574 (9)	−0.04221 (9)	0.73220 (6)	0.02490 (19)	

### 2-(4-Benzylbenzylidene)-6,7,8,9-tetrahydro-5H-benzo[7]annulen-5-one (III) Atomic displacement parameters (Å^2^)

**Table:**

	*U*^11^	*U*^22^	*U*^33^	*U*^12^	*U*^13^	*U*^23^
C1	0.0216 (5)	0.0216 (5)	0.0211 (5)	−0.0107 (4)	0.0026 (4)	−0.0097 (4)
C2	0.0206 (5)	0.0227 (5)	0.0199 (5)	−0.0118 (4)	0.0035 (4)	−0.0089 (4)
C3	0.0214 (5)	0.0210 (5)	0.0235 (5)	−0.0110 (4)	0.0009 (4)	−0.0069 (4)
C4	0.0233 (6)	0.0246 (5)	0.0255 (6)	−0.0122 (5)	0.0037 (4)	−0.0060 (4)
C5	0.0296 (6)	0.0267 (6)	0.0219 (5)	−0.0135 (5)	0.0058 (4)	−0.0094 (4)
C6	0.0298 (6)	0.0209 (5)	0.0224 (5)	−0.0128 (5)	0.0001 (4)	−0.0091 (4)
C7	0.0407 (7)	0.0254 (6)	0.0226 (6)	−0.0172 (5)	−0.0016 (5)	−0.0077 (5)
C8	0.0413 (7)	0.0218 (6)	0.0280 (6)	−0.0106 (5)	−0.0124 (5)	−0.0065 (5)
C9	0.0269 (6)	0.0248 (6)	0.0362 (6)	−0.0062 (5)	−0.0092 (5)	−0.0140 (5)
C10	0.0252 (6)	0.0254 (6)	0.0291 (6)	−0.0113 (5)	−0.0011 (4)	−0.0129 (5)
C11	0.0240 (5)	0.0197 (5)	0.0232 (5)	−0.0101 (4)	−0.0012 (4)	−0.0096 (4)
C12	0.0211 (5)	0.0232 (5)	0.0205 (5)	−0.0129 (4)	0.0036 (4)	−0.0091 (4)
C13	0.0208 (5)	0.0212 (5)	0.0219 (5)	−0.0097 (4)	0.0017 (4)	−0.0092 (4)
C14	0.0227 (5)	0.0230 (5)	0.0217 (5)	−0.0114 (4)	0.0006 (4)	−0.0046 (4)
C15	0.0226 (5)	0.0256 (5)	0.0243 (5)	−0.0144 (5)	0.0030 (4)	−0.0063 (4)
C16	0.0192 (5)	0.0221 (5)	0.0230 (5)	−0.0088 (4)	−0.0002 (4)	−0.0086 (4)
C17	0.0223 (5)	0.0197 (5)	0.0210 (5)	−0.0105 (4)	0.0011 (4)	−0.0055 (4)
C18	0.0214 (5)	0.0217 (5)	0.0235 (5)	−0.0122 (4)	0.0033 (4)	−0.0092 (4)
C19	0.0242 (6)	0.0305 (6)	0.0307 (6)	−0.0183 (5)	−0.0022 (5)	−0.0008 (5)
C20	0.0205 (5)	0.0242 (5)	0.0242 (5)	−0.0146 (4)	0.0018 (4)	−0.0033 (4)
C21	0.0288 (6)	0.0261 (6)	0.0255 (6)	−0.0174 (5)	0.0023 (4)	−0.0088 (4)
C22	0.0236 (6)	0.0217 (5)	0.0303 (6)	−0.0103 (5)	0.0030 (4)	−0.0062 (5)
C23	0.0236 (6)	0.0296 (6)	0.0250 (6)	−0.0153 (5)	−0.0030 (4)	−0.0009 (5)
C24	0.0355 (6)	0.0325 (6)	0.0231 (6)	−0.0209 (5)	0.0015 (5)	−0.0093 (5)
C25	0.0260 (6)	0.0224 (5)	0.0273 (6)	−0.0115 (5)	0.0056 (4)	−0.0085 (4)
O1	0.0258 (4)	0.0314 (4)	0.0242 (4)	−0.0182 (3)	0.0011 (3)	−0.0058 (3)
O2	0.0201 (4)	0.0269 (4)	0.0257 (4)	−0.0141 (3)	−0.0028 (3)	−0.0019 (3)

### 2-(4-Benzylbenzylidene)-6,7,8,9-tetrahydro-5H-benzo[7]annulen-5-one (III) Geometric parameters (Å, º)

**Table:**

C1—O1	1.2294 (12)	C13—C14	1.4001 (14)
C1—C2	1.4914 (14)	C13—C18	1.4042 (15)
C1—C11	1.5025 (14)	C14—C15	1.3895 (15)
C2—C12	1.3467 (15)	C14—H14	0.9500
C2—C3	1.5077 (14)	C15—C16	1.3933 (15)
C3—C4	1.5326 (15)	C15—H15	0.9500
C3—H3A	0.9900	C16—O2	1.3723 (12)
C3—H3B	0.9900	C16—C17	1.3936 (14)
C4—C5	1.5352 (15)	C17—C18	1.3774 (14)
C4—H4A	0.9900	C17—H17	0.9500
C4—H4B	0.9900	C18—H18	0.9500
C5—C6	1.5099 (15)	C19—O2	1.4392 (12)
C5—H5A	0.9900	C19—C20	1.4990 (15)
C5—H5B	0.9900	C19—H19A	0.9900
C6—C7	1.3945 (15)	C19—H19B	0.9900
C6—C11	1.4102 (15)	C20—C25	1.3916 (16)
C7—C8	1.3881 (17)	C20—C21	1.3925 (16)
C7—H7	0.9500	C21—C22	1.3849 (16)
C8—C9	1.3850 (18)	C21—H21	0.9500
C8—H8	0.9500	C22—C23	1.3850 (16)
C9—C10	1.3859 (16)	C22—H22	0.9500
C9—H9	0.9500	C23—C24	1.3850 (17)
C10—C11	1.3960 (15)	C23—H23	0.9500
C10—H10	0.9500	C24—C25	1.3871 (16)
C12—C13	1.4637 (14)	C24—H24	0.9500
C12—H12	0.9500	C25—H25	0.9500
			
O1—C1—C2	121.81 (9)	C13—C12—H12	114.0
O1—C1—C11	118.85 (9)	C14—C13—C18	116.95 (9)
C2—C1—C11	119.33 (9)	C14—C13—C12	125.98 (9)
C12—C2—C1	116.43 (9)	C18—C13—C12	117.06 (9)
C12—C2—C3	127.54 (9)	C15—C14—C13	121.79 (10)
C1—C2—C3	116.03 (9)	C15—C14—H14	119.1
C2—C3—C4	111.93 (9)	C13—C14—H14	119.1
C2—C3—H3A	109.2	C14—C15—C16	119.59 (10)
C4—C3—H3A	109.2	C14—C15—H15	120.2
C2—C3—H3B	109.2	C16—C15—H15	120.2
C4—C3—H3B	109.2	O2—C16—C15	124.70 (9)
H3A—C3—H3B	107.9	O2—C16—C17	115.57 (9)
C3—C4—C5	112.28 (9)	C15—C16—C17	119.73 (10)
C3—C4—H4A	109.1	C18—C17—C16	119.83 (10)
C5—C4—H4A	109.1	C18—C17—H17	120.1
C3—C4—H4B	109.1	C16—C17—H17	120.1
C5—C4—H4B	109.1	C17—C18—C13	122.01 (9)
H4A—C4—H4B	107.9	C17—C18—H18	119.0
C6—C5—C4	114.06 (9)	C13—C18—H18	119.0
C6—C5—H5A	108.7	O2—C19—C20	108.38 (8)
C4—C5—H5A	108.7	O2—C19—H19A	110.0
C6—C5—H5B	108.7	C20—C19—H19A	110.0
C4—C5—H5B	108.7	O2—C19—H19B	110.0
H5A—C5—H5B	107.6	C20—C19—H19B	110.0
C7—C6—C11	118.30 (10)	H19A—C19—H19B	108.4
C7—C6—C5	120.45 (10)	C25—C20—C21	118.80 (10)
C11—C6—C5	121.17 (9)	C25—C20—C19	121.35 (10)
C8—C7—C6	121.25 (11)	C21—C20—C19	119.83 (10)
C8—C7—H7	119.4	C22—C21—C20	120.46 (10)
C6—C7—H7	119.4	C22—C21—H21	119.8
C9—C8—C7	120.17 (10)	C20—C21—H21	119.8
C9—C8—H8	119.9	C21—C22—C23	120.37 (10)
C7—C8—H8	119.9	C21—C22—H22	119.8
C8—C9—C10	119.62 (11)	C23—C22—H22	119.8
C8—C9—H9	120.2	C24—C23—C22	119.60 (10)
C10—C9—H9	120.2	C24—C23—H23	120.2
C9—C10—C11	120.72 (11)	C22—C23—H23	120.2
C9—C10—H10	119.6	C23—C24—C25	120.12 (10)
C11—C10—H10	119.6	C23—C24—H24	119.9
C10—C11—C6	119.92 (10)	C25—C24—H24	119.9
C10—C11—C1	117.49 (9)	C24—C25—C20	120.64 (10)
C6—C11—C1	122.50 (9)	C24—C25—H25	119.7
C2—C12—C13	132.05 (9)	C20—C25—H25	119.7
C2—C12—H12	114.0	C16—O2—C19	116.40 (8)
			
O1—C1—C2—C12	20.98 (15)	C3—C2—C12—C13	0.73 (19)
C11—C1—C2—C12	−159.81 (9)	C2—C12—C13—C14	−17.70 (19)
O1—C1—C2—C3	−159.38 (10)	C2—C12—C13—C18	163.17 (11)
C11—C1—C2—C3	19.82 (13)	C18—C13—C14—C15	−2.86 (16)
C12—C2—C3—C4	95.36 (13)	C12—C13—C14—C15	178.01 (10)
C1—C2—C3—C4	−84.23 (11)	C13—C14—C15—C16	0.48 (16)
C2—C3—C4—C5	43.11 (12)	C14—C15—C16—O2	−178.18 (10)
C3—C4—C5—C6	45.09 (12)	C14—C15—C16—C17	1.83 (16)
C4—C5—C6—C7	112.75 (11)	O2—C16—C17—C18	178.36 (9)
C4—C5—C6—C11	−70.46 (13)	C15—C16—C17—C18	−1.65 (16)
C11—C6—C7—C8	0.23 (16)	C16—C17—C18—C13	−0.86 (16)
C5—C6—C7—C8	177.11 (10)	C14—C13—C18—C17	3.06 (15)
C6—C7—C8—C9	−0.60 (17)	C12—C13—C18—C17	−177.73 (9)
C7—C8—C9—C10	0.04 (16)	O2—C19—C20—C25	−102.31 (11)
C8—C9—C10—C11	0.89 (16)	O2—C19—C20—C21	79.43 (12)
C9—C10—C11—C6	−1.26 (15)	C25—C20—C21—C22	0.41 (15)
C9—C10—C11—C1	−177.85 (9)	C19—C20—C21—C22	178.72 (9)
C7—C6—C11—C10	0.69 (15)	C20—C21—C22—C23	−1.38 (16)
C5—C6—C11—C10	−176.17 (9)	C21—C22—C23—C24	1.27 (16)
C7—C6—C11—C1	177.10 (9)	C22—C23—C24—C25	−0.20 (16)
C5—C6—C11—C1	0.25 (15)	C23—C24—C25—C20	−0.77 (16)
O1—C1—C11—C10	32.34 (14)	C21—C20—C25—C24	0.67 (16)
C2—C1—C11—C10	−146.89 (10)	C19—C20—C25—C24	−177.61 (10)
O1—C1—C11—C6	−144.16 (10)	C15—C16—O2—C19	0.07 (15)
C2—C1—C11—C6	36.61 (14)	C17—C16—O2—C19	−179.94 (9)
C1—C2—C12—C13	−179.68 (10)	C20—C19—O2—C16	−179.68 (9)

### 2-(4-Benzylbenzylidene)-6,7,8,9-tetrahydro-5H-benzo[7]annulen-5-one (III) Hydrogen-bond geometry (Å, º)

**Table:**

*D*—H···*A*	*D*—H	H···*A*	*D*···*A*	*D*—H···*A*
C15—H15···O1^i^	0.95	2.40	3.3477 (13)	176
C18—H18···*Cg*3^ii^	0.95	2.64	3.5147 (13)	153

Symmetry codes: (i) *x*+1, *y*, *z*; (ii) *x*−1, *y*, *z*.

### 2-(4-Chlorobenzylidene)-6,7,8,9-tetrahydro-5H-benzo[7]annulen-5-one (IV) Crystal data

**Table:**

C_18_H_15_ClO	*F*(000) = 592
*M**_r_* = 282.75	*D*_x_ = 1.326 Mg m^−^^3^
Monoclinic, *P*2_1_/*n*	Cu *K*α radiation, λ = 1.54184 Å
*a* = 10.6273 (5) Å	Cell parameters from 4926 reflections
*b* = 11.6191 (4) Å	θ = 4.8–70.0°
*c* = 12.1114 (5) Å	µ = 2.31 mm^−^^1^
β = 108.777 (4)°	*T* = 100 K
*V* = 1415.92 (11) Å^3^	Plate, colourless
*Z* = 4	0.28 × 0.20 × 0.03 mm

### 2-(4-Chlorobenzylidene)-6,7,8,9-tetrahydro-5H-benzo[7]annulen-5-one (IV) Data collection

**Table:**

XtaLAB AFC11 (RCD3): quarter-chi single CCD diffractometer	2568 independent reflections
Radiation source: Rotating-anode X-ray tube	2203 reflections with *I* > 2σ(*I*)
Mirror monochromator	*R*_int_ = 0.073
ω scans	θ_max_ = 68.2°, θ_min_ = 4.8°
Absorption correction: multi-scan (CrysAlis PRO; Rigaku, 2017)	*h* = −12→11
*T*_min_ = 0.722, *T*_max_ = 1.000	*k* = −13→13
11747 measured reflections	*l* = −14→13

### 2-(4-Chlorobenzylidene)-6,7,8,9-tetrahydro-5H-benzo[7]annulen-5-one (IV) Refinement

**Table:**

Refinement on *F*^2^	Primary atom site location: structure-invariant direct methods
Least-squares matrix: full	Hydrogen site location: inferred from neighbouring sites
*R*[*F*^2^ > 2σ(*F*^2^)] = 0.055	H-atom parameters constrained
*wR*(*F*^2^) = 0.165	*w* = 1/[σ^2^(*F*_o_^2^) + (0.1145*P*)^2^ + 0.0344*P*] where *P* = (*F*_o_^2^ + 2*F*_c_^2^)/3
*S* = 1.11	(Δ/σ)_max_ = 0.001
2568 reflections	Δρ_max_ = 0.32 e Å^−^^3^
181 parameters	Δρ_min_ = −0.41 e Å^−^^3^
0 restraints	

### 2-(4-Chlorobenzylidene)-6,7,8,9-tetrahydro-5H-benzo[7]annulen-5-one (IV) Special details

**Table:**

Geometry. All esds (except the esd in the dihedral angle between two l.s. planes) are estimated using the full covariance matrix. The cell esds are taken into account individually in the estimation of esds in distances, angles and torsion angles; correlations between esds in cell parameters are only used when they are defined by crystal symmetry. An approximate (isotropic) treatment of cell esds is used for estimating esds involving l.s. planes.

### 2-(4-Chlorobenzylidene)-6,7,8,9-tetrahydro-5H-benzo[7]annulen-5-one (IV) Fractional atomic coordinates and isotropic or equivalent isotropic displacement parameters (Å^2^)

**Table:**

	*x*	*y*	*z*	*U*_iso_*/*U*_eq_	
C1	0.2086 (2)	0.65193 (16)	0.58219 (17)	0.0292 (4)	
C2	0.34239 (19)	0.67189 (16)	0.67264 (17)	0.0292 (4)	
C3	0.34206 (19)	0.73875 (16)	0.77902 (17)	0.0311 (5)	
H3A	0.4244	0.7207	0.8436	0.037*	
H3B	0.2658	0.7131	0.8029	0.037*	
C4	0.3330 (2)	0.86976 (16)	0.76058 (18)	0.0333 (5)	
H4A	0.4225	0.9004	0.7685	0.040*	
H4B	0.3028	0.9057	0.8218	0.040*	
C5	0.2366 (2)	0.90247 (17)	0.64003 (18)	0.0323 (5)	
H5A	0.2215	0.9866	0.6372	0.039*	
H5B	0.2775	0.8829	0.5796	0.039*	
C6	0.1047 (2)	0.84132 (17)	0.61283 (17)	0.0301 (5)	
C7	−0.0085 (2)	0.90160 (19)	0.61159 (17)	0.0353 (5)	
H7	−0.0024	0.9820	0.6268	0.042*	
C8	−0.1309 (2)	0.84680 (19)	0.58856 (19)	0.0367 (5)	
H8	−0.2071	0.8896	0.5885	0.044*	
C9	−0.1412 (2)	0.72966 (19)	0.56571 (17)	0.0361 (5)	
H9	−0.2242	0.6917	0.5506	0.043*	
C10	−0.0297 (2)	0.66851 (18)	0.56509 (17)	0.0330 (5)	
H10	−0.0369	0.5884	0.5486	0.040*	
C11	0.09310 (19)	0.72291 (16)	0.58837 (16)	0.0294 (5)	
C12	0.4476 (2)	0.62505 (16)	0.65047 (18)	0.0317 (5)	
H12	0.4264	0.5825	0.5798	0.038*	
C13	0.5893 (2)	0.62893 (16)	0.71748 (18)	0.0303 (5)	
C14	0.6506 (2)	0.71561 (17)	0.79718 (18)	0.0334 (5)	
H14	0.5983	0.7765	0.8116	0.040*	
C15	0.7861 (2)	0.71376 (17)	0.85517 (18)	0.0339 (5)	
H15	0.8263	0.7730	0.9090	0.041*	
C16	0.8629 (2)	0.62534 (17)	0.83450 (19)	0.0341 (5)	
C17	0.8062 (2)	0.53886 (18)	0.75589 (19)	0.0391 (5)	
H17	0.8593	0.4783	0.7421	0.047*	
C18	0.6709 (2)	0.54195 (18)	0.69764 (18)	0.0360 (5)	
H18	0.6321	0.4833	0.6425	0.043*	
O1	0.19203 (14)	0.57880 (12)	0.50600 (12)	0.0347 (4)	
Cl1	1.03346 (5)	0.62611 (5)	0.90563 (5)	0.0469 (3)	

### 2-(4-Chlorobenzylidene)-6,7,8,9-tetrahydro-5H-benzo[7]annulen-5-one (IV) Atomic displacement parameters (Å^2^)

**Table:**

	*U*^11^	*U*^22^	*U*^33^	*U*^12^	*U*^13^	*U*^23^
C1	0.0308 (11)	0.0267 (9)	0.0297 (10)	−0.0017 (8)	0.0091 (8)	0.0023 (8)
C2	0.0292 (10)	0.0258 (9)	0.0307 (10)	−0.0012 (7)	0.0068 (8)	0.0018 (7)
C3	0.0273 (10)	0.0325 (10)	0.0309 (10)	0.0007 (8)	0.0056 (8)	−0.0006 (8)
C4	0.0274 (11)	0.0321 (11)	0.0371 (12)	−0.0021 (8)	0.0056 (9)	−0.0064 (8)
C5	0.0308 (11)	0.0277 (9)	0.0362 (11)	−0.0004 (8)	0.0076 (9)	−0.0006 (8)
C6	0.0289 (11)	0.0296 (10)	0.0295 (10)	−0.0005 (8)	0.0063 (8)	0.0004 (8)
C7	0.0330 (11)	0.0362 (11)	0.0330 (11)	0.0017 (9)	0.0056 (9)	−0.0004 (9)
C8	0.0286 (11)	0.0469 (12)	0.0328 (11)	0.0045 (9)	0.0074 (9)	−0.0001 (9)
C9	0.0289 (11)	0.0455 (12)	0.0312 (11)	−0.0047 (9)	0.0059 (9)	0.0015 (9)
C10	0.0323 (11)	0.0344 (11)	0.0295 (10)	−0.0043 (8)	0.0062 (8)	0.0008 (8)
C11	0.0280 (11)	0.0318 (10)	0.0263 (10)	0.0004 (8)	0.0058 (8)	0.0017 (7)
C12	0.0350 (12)	0.0274 (10)	0.0303 (11)	−0.0015 (8)	0.0073 (9)	0.0008 (7)
C13	0.0306 (11)	0.0317 (10)	0.0284 (10)	0.0020 (8)	0.0090 (9)	0.0025 (8)
C14	0.0296 (11)	0.0314 (10)	0.0384 (11)	0.0005 (8)	0.0099 (9)	−0.0030 (8)
C15	0.0325 (11)	0.0338 (10)	0.0350 (11)	−0.0023 (8)	0.0101 (9)	−0.0016 (8)
C16	0.0281 (11)	0.0383 (11)	0.0356 (12)	0.0005 (8)	0.0099 (9)	0.0045 (8)
C17	0.0339 (11)	0.0373 (12)	0.0465 (13)	0.0049 (9)	0.0134 (10)	−0.0025 (9)
C18	0.0334 (11)	0.0344 (11)	0.0397 (12)	−0.0005 (8)	0.0110 (9)	−0.0059 (8)
O1	0.0348 (8)	0.0318 (8)	0.0354 (8)	−0.0029 (6)	0.0083 (6)	−0.0060 (6)
Cl1	0.0268 (4)	0.0552 (4)	0.0545 (4)	0.0032 (2)	0.0071 (3)	0.0000 (2)

### 2-(4-Chlorobenzylidene)-6,7,8,9-tetrahydro-5H-benzo[7]annulen-5-one (IV) Geometric parameters (Å, º)

**Table:**

C1—O1	1.225 (2)	C8—H8	0.9500
C1—C11	1.501 (3)	C9—C10	1.384 (3)
C1—C2	1.507 (3)	C9—H9	0.9500
C2—C12	1.346 (3)	C10—C11	1.395 (3)
C2—C3	1.506 (3)	C10—H10	0.9500
C3—C4	1.537 (3)	C12—C13	1.464 (3)
C3—H3A	0.9900	C12—H12	0.9500
C3—H3B	0.9900	C13—C18	1.402 (3)
C4—C5	1.537 (3)	C13—C14	1.402 (3)
C4—H4A	0.9900	C14—C15	1.385 (3)
C4—H4B	0.9900	C14—H14	0.9500
C5—C6	1.510 (3)	C15—C16	1.384 (3)
C5—H5A	0.9900	C15—H15	0.9500
C5—H5B	0.9900	C16—C17	1.382 (3)
C6—C7	1.388 (3)	C16—Cl1	1.739 (2)
C6—C11	1.404 (3)	C17—C18	1.383 (3)
C7—C8	1.393 (3)	C17—H17	0.9500
C7—H7	0.9500	C18—H18	0.9500
C8—C9	1.386 (3)		
			
O1—C1—C11	119.82 (18)	C7—C8—H8	120.1
O1—C1—C2	121.84 (18)	C10—C9—C8	119.48 (19)
C11—C1—C2	118.33 (16)	C10—C9—H9	120.3
C12—C2—C3	127.61 (18)	C8—C9—H9	120.3
C12—C2—C1	116.26 (17)	C9—C10—C11	120.90 (19)
C3—C2—C1	116.09 (16)	C9—C10—H10	119.6
C2—C3—C4	113.82 (16)	C11—C10—H10	119.6
C2—C3—H3A	108.8	C10—C11—C6	119.99 (18)
C4—C3—H3A	108.8	C10—C11—C1	117.91 (17)
C2—C3—H3B	108.8	C6—C11—C1	122.07 (17)
C4—C3—H3B	108.8	C2—C12—C13	130.51 (18)
H3A—C3—H3B	107.7	C2—C12—H12	114.7
C3—C4—C5	111.98 (16)	C13—C12—H12	114.7
C3—C4—H4A	109.2	C18—C13—C14	117.39 (19)
C5—C4—H4A	109.2	C18—C13—C12	117.75 (18)
C3—C4—H4B	109.2	C14—C13—C12	124.81 (17)
C5—C4—H4B	109.2	C15—C14—C13	120.99 (18)
H4A—C4—H4B	107.9	C15—C14—H14	119.5
C6—C5—C4	112.27 (17)	C13—C14—H14	119.5
C6—C5—H5A	109.1	C16—C15—C14	119.82 (19)
C4—C5—H5A	109.1	C16—C15—H15	120.1
C6—C5—H5B	109.1	C14—C15—H15	120.1
C4—C5—H5B	109.1	C17—C16—C15	120.9 (2)
H5A—C5—H5B	107.9	C17—C16—Cl1	119.91 (16)
C7—C6—C11	118.35 (19)	C15—C16—Cl1	119.19 (17)
C7—C6—C5	120.36 (18)	C16—C17—C18	118.90 (19)
C11—C6—C5	121.30 (18)	C16—C17—H17	120.6
C6—C7—C8	121.4 (2)	C18—C17—H17	120.6
C6—C7—H7	119.3	C17—C18—C13	122.0 (2)
C8—C7—H7	119.3	C17—C18—H18	119.0
C9—C8—C7	119.8 (2)	C13—C18—H18	119.0
C9—C8—H8	120.1		
			
O1—C1—C2—C12	14.6 (3)	C5—C6—C11—C1	−3.0 (3)
C11—C1—C2—C12	−166.52 (17)	O1—C1—C11—C10	38.8 (3)
O1—C1—C2—C3	−163.47 (18)	C2—C1—C11—C10	−140.10 (18)
C11—C1—C2—C3	15.4 (2)	O1—C1—C11—C6	−139.2 (2)
C12—C2—C3—C4	101.3 (2)	C2—C1—C11—C6	42.0 (3)
C1—C2—C3—C4	−80.8 (2)	C3—C2—C12—C13	−3.2 (3)
C2—C3—C4—C5	39.8 (2)	C1—C2—C12—C13	178.97 (18)
C3—C4—C5—C6	48.7 (2)	C2—C12—C13—C18	157.7 (2)
C4—C5—C6—C7	108.5 (2)	C2—C12—C13—C14	−24.7 (3)
C4—C5—C6—C11	−71.4 (2)	C18—C13—C14—C15	−1.1 (3)
C11—C6—C7—C8	1.0 (3)	C12—C13—C14—C15	−178.65 (19)
C5—C6—C7—C8	−178.97 (19)	C13—C14—C15—C16	0.0 (3)
C6—C7—C8—C9	−0.3 (3)	C14—C15—C16—C17	0.5 (3)
C7—C8—C9—C10	−0.5 (3)	C14—C15—C16—Cl1	178.57 (16)
C8—C9—C10—C11	0.7 (3)	C15—C16—C17—C18	0.1 (3)
C9—C10—C11—C6	0.0 (3)	Cl1—C16—C17—C18	−178.02 (16)
C9—C10—C11—C1	−177.98 (18)	C16—C17—C18—C13	−1.2 (3)
C7—C6—C11—C10	−0.8 (3)	C14—C13—C18—C17	1.6 (3)
C5—C6—C11—C10	179.14 (17)	C12—C13—C18—C17	179.39 (18)
C7—C6—C11—C1	177.07 (17)		

### 2-(4-Chlorobenzylidene)-6,7,8,9-tetrahydro-5H-benzo[7]annulen-5-one (IV) Hydrogen-bond geometry (Å, º)

**Table:**

*D*—H···*A*	*D*—H	H···*A*	*D*···*A*	*D*—H···*A*
C10—H10···O1^i^	0.95	2.50	3.319 (2)	145
C3—H3*A*···*Cg*1^ii^	0.99	2.83	3.572 (2)	132

Symmetry codes: (i) −*x*, −*y*+1, −*z*+1; (ii) *x*−1/2, −*y*+3/2, *z*+1/2.

### 6-(4-Cyanobenzylidene)-6,7,8,9-tetrahydro-5H-benzo[7]annulen-5-one (V) Crystal data

**Table:**

C_19_H_15_NO	*F*(000) = 576
*M**_r_* = 273.32	*D*_x_ = 1.321 Mg m^−^^3^
Monoclinic, *P*2_1_/*c*	Cu *K*α radiation, λ = 1.54184 Å
*a* = 12.4725 (4) Å	Cell parameters from 3885 reflections
*b* = 7.1718 (2) Å	θ = 5.7–69.4°
*c* = 15.9983 (5) Å	µ = 0.64 mm^−^^1^
β = 106.120 (3)°	*T* = 100 K
*V* = 1374.79 (8) Å^3^	Block, colourless
*Z* = 4	0.17 × 0.10 × 0.03 mm

### 6-(4-Cyanobenzylidene)-6,7,8,9-tetrahydro-5H-benzo[7]annulen-5-one (V) Data collection

**Table:**

XtaLAB AFC11 (RCD3): quarter-chi single CCD diffractometer	2511 independent reflections
Radiation source: Rotating-anode X-ray tube	2302 reflections with *I* > 2σ(*I*)
Mirror monochromator	*R*_int_ = 0.059
ω scans	θ_max_ = 68.2°, θ_min_ = 3.7°
Absorption correction: gaussian (CrysAlis PRO; Rigaku, 2017)	*h* = −14→15
*T*_min_ = 0.895, *T*_max_ = 1.000	*k* = −7→8
9732 measured reflections	*l* = −19→19

### 6-(4-Cyanobenzylidene)-6,7,8,9-tetrahydro-5H-benzo[7]annulen-5-one (V) Refinement

**Table:**

Refinement on *F*^2^	Primary atom site location: structure-invariant direct methods
Least-squares matrix: full	Hydrogen site location: inferred from neighbouring sites
*R*[*F*^2^ > 2σ(*F*^2^)] = 0.068	H-atom parameters constrained
*wR*(*F*^2^) = 0.181	*w* = 1/[σ^2^(*F*_o_^2^) + (0.1396*P*)^2^ + 0.1712*P*] where *P* = (*F*_o_^2^ + 2*F*_c_^2^)/3
*S* = 1.06	(Δ/σ)_max_ < 0.001
2511 reflections	Δρ_max_ = 0.49 e Å^−^^3^
190 parameters	Δρ_min_ = −0.32 e Å^−^^3^
0 restraints	

### 6-(4-Cyanobenzylidene)-6,7,8,9-tetrahydro-5H-benzo[7]annulen-5-one (V) Special details

**Table:**

Geometry. All esds (except the esd in the dihedral angle between two l.s. planes) are estimated using the full covariance matrix. The cell esds are taken into account individually in the estimation of esds in distances, angles and torsion angles; correlations between esds in cell parameters are only used when they are defined by crystal symmetry. An approximate (isotropic) treatment of cell esds is used for estimating esds involving l.s. planes.

### 6-(4-Cyanobenzylidene)-6,7,8,9-tetrahydro-5H-benzo[7]annulen-5-one (V) Fractional atomic coordinates and isotropic or equivalent isotropic displacement parameters (Å^2^)

**Table:**

	*x*	*y*	*z*	*U*_iso_*/*U*_eq_	
C1	0.34825 (12)	0.5911 (2)	0.45058 (9)	0.0284 (4)	
C2	0.29525 (12)	0.4044 (2)	0.42080 (9)	0.0275 (4)	
C3	0.32674 (12)	0.3153 (2)	0.34549 (9)	0.0279 (4)	
H3A	0.3150	0.1790	0.3474	0.033*	
H3B	0.4073	0.3366	0.3529	0.033*	
C4	0.26091 (13)	0.3882 (2)	0.25527 (10)	0.0292 (4)	
H4A	0.3080	0.3783	0.2148	0.035*	
H4B	0.1941	0.3093	0.2323	0.035*	
C5	0.22444 (13)	0.5911 (2)	0.25887 (10)	0.0296 (4)	
H5A	0.1850	0.6337	0.1994	0.036*	
H5B	0.1717	0.5993	0.2950	0.036*	
C6	0.32277 (12)	0.7168 (2)	0.29642 (9)	0.0279 (4)	
C7	0.35894 (13)	0.8371 (2)	0.24193 (10)	0.0304 (4)	
H7	0.3192	0.8421	0.1820	0.037*	
C8	0.45193 (14)	0.9506 (2)	0.27303 (11)	0.0319 (4)	
H8	0.4759	1.0306	0.2344	0.038*	
C9	0.50973 (13)	0.9462 (2)	0.36109 (11)	0.0318 (4)	
H9	0.5734	1.0232	0.3829	0.038*	
C10	0.47389 (13)	0.8293 (2)	0.41635 (10)	0.0305 (4)	
H10	0.5128	0.8279	0.4765	0.037*	
C11	0.38136 (12)	0.7129 (2)	0.38553 (10)	0.0279 (4)	
C12	0.23202 (13)	0.3273 (2)	0.46664 (10)	0.0294 (4)	
H12	0.2248	0.3944	0.5160	0.035*	
C13	0.17236 (13)	0.1477 (2)	0.44785 (10)	0.0284 (4)	
C14	0.11644 (13)	0.0939 (2)	0.36261 (10)	0.0304 (4)	
H14	0.1162	0.1752	0.3157	0.036*	
C15	0.06168 (13)	−0.0752 (2)	0.34565 (10)	0.0304 (4)	
H15	0.0252	−0.1102	0.2874	0.036*	
C16	0.05996 (12)	−0.1944 (2)	0.41403 (10)	0.0288 (4)	
C17	0.11421 (14)	−0.1427 (2)	0.49975 (10)	0.0327 (4)	
H17	0.1138	−0.2237	0.5466	0.039*	
C18	0.16847 (13)	0.0273 (2)	0.51552 (10)	0.0320 (4)	
H18	0.2040	0.0630	0.5738	0.038*	
C19	−0.00015 (13)	−0.3676 (2)	0.39548 (10)	0.0308 (4)	
N1	−0.05047 (12)	−0.5036 (2)	0.37711 (9)	0.0374 (4)	
O1	0.36770 (11)	0.63971 (17)	0.52639 (7)	0.0376 (4)	

### 6-(4-Cyanobenzylidene)-6,7,8,9-tetrahydro-5H-benzo[7]annulen-5-one (V) Atomic displacement parameters (Å^2^)

**Table:**

	*U*^11^	*U*^22^	*U*^33^	*U*^12^	*U*^13^	*U*^23^
C1	0.0239 (8)	0.0341 (9)	0.0233 (8)	0.0023 (6)	0.0002 (6)	−0.0017 (6)
C2	0.0235 (7)	0.0299 (8)	0.0234 (8)	0.0018 (6)	−0.0028 (6)	0.0020 (6)
C3	0.0228 (7)	0.0306 (8)	0.0273 (8)	0.0001 (6)	0.0020 (6)	−0.0004 (6)
C4	0.0263 (8)	0.0345 (9)	0.0243 (8)	−0.0036 (6)	0.0030 (6)	−0.0032 (6)
C5	0.0256 (8)	0.0356 (9)	0.0231 (8)	−0.0009 (6)	−0.0009 (6)	0.0006 (6)
C6	0.0248 (8)	0.0304 (8)	0.0264 (8)	0.0042 (6)	0.0034 (6)	−0.0011 (6)
C7	0.0278 (8)	0.0311 (8)	0.0307 (9)	0.0048 (6)	0.0054 (7)	0.0001 (6)
C8	0.0314 (8)	0.0286 (8)	0.0375 (9)	0.0031 (6)	0.0122 (7)	0.0008 (6)
C9	0.0244 (8)	0.0305 (8)	0.0391 (9)	−0.0001 (6)	0.0065 (7)	−0.0044 (7)
C10	0.0254 (8)	0.0307 (8)	0.0317 (9)	0.0019 (6)	0.0016 (6)	−0.0038 (6)
C11	0.0235 (7)	0.0288 (8)	0.0292 (8)	0.0015 (6)	0.0035 (6)	−0.0021 (6)
C12	0.0272 (8)	0.0323 (8)	0.0243 (8)	0.0018 (6)	−0.0002 (6)	−0.0001 (6)
C13	0.0236 (8)	0.0321 (9)	0.0277 (8)	0.0015 (6)	0.0042 (6)	−0.0011 (6)
C14	0.0274 (8)	0.0340 (9)	0.0257 (8)	−0.0011 (6)	0.0009 (6)	0.0060 (6)
C15	0.0263 (8)	0.0354 (9)	0.0248 (8)	−0.0013 (6)	−0.0006 (6)	0.0005 (6)
C16	0.0239 (7)	0.0300 (8)	0.0294 (8)	0.0004 (6)	0.0025 (6)	0.0006 (6)
C17	0.0332 (8)	0.0359 (9)	0.0256 (8)	−0.0014 (7)	0.0027 (7)	0.0045 (6)
C18	0.0312 (8)	0.0371 (9)	0.0245 (8)	−0.0022 (7)	0.0024 (6)	−0.0005 (6)
C19	0.0300 (8)	0.0339 (9)	0.0259 (8)	0.0023 (7)	0.0033 (6)	0.0036 (6)
N1	0.0377 (8)	0.0359 (9)	0.0338 (8)	−0.0053 (6)	0.0018 (6)	0.0021 (6)
O1	0.0426 (7)	0.0412 (7)	0.0259 (6)	−0.0094 (5)	0.0042 (5)	−0.0047 (5)

### 6-(4-Cyanobenzylidene)-6,7,8,9-tetrahydro-5H-benzo[7]annulen-5-one (V) Geometric parameters (Å, º)

**Table:**

C1—O1	1.2199 (19)	C8—H8	0.9500
C1—C11	1.502 (2)	C9—C10	1.380 (2)
C1—C2	1.510 (2)	C9—H9	0.9500
C2—C12	1.337 (2)	C10—C11	1.399 (2)
C2—C3	1.509 (2)	C10—H10	0.9500
C3—C4	1.540 (2)	C12—C13	1.476 (2)
C3—H3A	0.9900	C12—H12	0.9500
C3—H3B	0.9900	C13—C18	1.396 (2)
C4—C5	1.531 (2)	C13—C14	1.402 (2)
C4—H4A	0.9900	C14—C15	1.381 (2)
C4—H4B	0.9900	C14—H14	0.9500
C5—C6	1.506 (2)	C15—C16	1.393 (2)
C5—H5A	0.9900	C15—H15	0.9500
C5—H5B	0.9900	C16—C17	1.400 (2)
C6—C7	1.388 (2)	C16—C19	1.439 (2)
C6—C11	1.410 (2)	C17—C18	1.383 (2)
C7—C8	1.391 (2)	C17—H17	0.9500
C7—H7	0.9500	C18—H18	0.9500
C8—C9	1.393 (2)	C19—N1	1.153 (2)
			
O1—C1—C11	120.38 (14)	C9—C8—H8	120.2
O1—C1—C2	121.05 (14)	C10—C9—C8	119.53 (15)
C11—C1—C2	118.52 (13)	C10—C9—H9	120.2
C12—C2—C3	125.92 (15)	C8—C9—H9	120.2
C12—C2—C1	117.85 (14)	C9—C10—C11	121.29 (14)
C3—C2—C1	116.04 (13)	C9—C10—H10	119.4
C2—C3—C4	114.54 (13)	C11—C10—H10	119.4
C2—C3—H3A	108.6	C10—C11—C6	119.32 (14)
C4—C3—H3A	108.6	C10—C11—C1	117.50 (14)
C2—C3—H3B	108.6	C6—C11—C1	123.17 (14)
C4—C3—H3B	108.6	C2—C12—C13	126.19 (14)
H3A—C3—H3B	107.6	C2—C12—H12	116.9
C5—C4—C3	111.96 (12)	C13—C12—H12	116.9
C5—C4—H4A	109.2	C18—C13—C14	117.97 (14)
C3—C4—H4A	109.2	C18—C13—C12	120.39 (14)
C5—C4—H4B	109.2	C14—C13—C12	121.63 (14)
C3—C4—H4B	109.2	C15—C14—C13	121.18 (14)
H4A—C4—H4B	107.9	C15—C14—H14	119.4
C6—C5—C4	111.51 (12)	C13—C14—H14	119.4
C6—C5—H5A	109.3	C14—C15—C16	119.93 (14)
C4—C5—H5A	109.3	C14—C15—H15	120.0
C6—C5—H5B	109.3	C16—C15—H15	120.0
C4—C5—H5B	109.3	C15—C16—C17	119.88 (15)
H5A—C5—H5B	108.0	C15—C16—C19	119.24 (14)
C7—C6—C11	118.67 (14)	C17—C16—C19	120.86 (14)
C7—C6—C5	119.49 (13)	C18—C17—C16	119.40 (15)
C11—C6—C5	121.83 (14)	C18—C17—H17	120.3
C6—C7—C8	121.55 (15)	C16—C17—H17	120.3
C6—C7—H7	119.2	C17—C18—C13	121.62 (14)
C8—C7—H7	119.2	C17—C18—H18	119.2
C7—C8—C9	119.64 (15)	C13—C18—H18	119.2
C7—C8—H8	120.2	N1—C19—C16	177.09 (16)

### 6-(4-Cyanobenzylidene)-6,7,8,9-tetrahydro-5H-benzo[7]annulen-5-one (V) Hydrogen-bond geometry (Å, º)

**Table:**

*D*—H···*A*	*D*—H	H···*A*	*D*···*A*	*D*—H···*A*
C17—H17···N1^i^	0.95	2.54	3.438 (2)	157
C3—H3*A*···*Cg*1^ii^	0.99	2.84	3.6730 (16)	142
C8—H8···*Cg*1^iii^	0.95	2.88	3.7868 (17)	161

Symmetry codes: (i) −*x*, −*y*−1, −*z*+1; (ii) *x*, *y*−1, *z*; (iii) −*x*+1, *y*+1/2, −*z*+1/2.
